# Agricultural Practices and Environmental Factors Drive Microbial Communities in the Mezcal-Producing *Agave angustifolia* Haw

**DOI:** 10.1007/s00248-025-02496-2

**Published:** 2025-01-30

**Authors:** Gonzalo Contreras-Negrete, Alfonso Valiente-Banuet, Francisco Molina-Freaner, Laila P. Partida-Martínez, Antonio Hernández-López

**Affiliations:** 1 Ciencias Agrogenómicas, Escuela Nacional de Estudios Superiores Unidad León, Universidad Nacional Autónoma de México, León, Guanajuato México; 2 Departamento de Ecología de La Biodiversidad, Instituto de Ecología, Universidad Nacional Autónoma de México, Mexico City, México; 3 Departamento de Ingeniería Genética, Centro de Investigación y de Estudios Avanzados del Instituto Politécnico Nacional, Unidad Irapuato, Irapuato, México; 4 Departamento de Ecología de la Biodiversidad, Instituto de Ecología, Universidad Nacional Autónoma de México, Hermosillo, Sonora México

**Keywords:** Agricultural practices, Microbiome, Domestication, Agave, Spirits

## Abstract

Mezcal, a traditional Mexican alcoholic beverage, has been a vital source of livelihood for indigenous and rural communities for centuries. However, increasing international demand is exerting pressure on natural resources and encouraging intensive agricultural practices. This study investigates the impact of management practices (wild, traditional, and conventional) and environmental factors on the microbial communities associated with *Agave angustifolia*, a key species in mezcal production. High-throughput sequencing of the 16S rRNA and ITS2 gene regions revealed distinct prokaryotic and fungal community structures across different plant compartments (endosphere, episphere, and soil), identifying 8214 prokaryotic and 7459 fungal ASVs. Core microbial communities were dominated by Proteobacteria, Actinobacteria, Ascomycota, and Basidiomycota. Alpha diversity analyses showed significant increases in prokaryotic diversity from the endosphere to soil, while fungal diversity remained stable. Notably, conventional management practices were associated with reductions in beneficial microbial taxa. Environmental factors such as precipitation and temperature significantly influenced microbial diversity and composition, especially in the rhizosphere. Beta diversity patterns underscored the strong impact of plant compartment, with management practices and aridity further shaping microbial communities. These results reveal the intricate interactions between management practices, environmental conditions, and microbial diversity, providing valuable insights for the sustainable cultivation of *A. angustifolia*.

**Supplementary Information:**

The online version contains supplementary material available at 10.1007/s00248-025-02496-2.

## Introduction

Plant-associated microorganisms play a central role in vital functions such as germination, growth, and development of plants [1]. For plant species, particularly those with widespread distribution, microbial communities are influenced by a combination of intrinsic factors (such as genotype and phenotype) and extrinsic factors (including climate, geography, soil type, and seasons). Additionally, the type of holobiont sample crucially shapes microbial diversity and composition under natural conditions [2–5]. In contrast, plants subjected to artificial selection often exhibit significant changes in microbiome diversity and composition. These changes are frequently associated with morphological and physiological alterations in managed genotypes (e.g., *Phaseolus* spp., barley, wheat, soybean) [6–8], particularly in root traits such as the root endosphere and rhizosphere [6, 7, 9].

Positive interactions between the host and associated microbiome (*eubiosis*) for both prokaryotic and fungal communities significantly enhance beneficial interactions such as diazotrophic functions, CO oxidation, and nutrient availability, as well as tolerance to drought, salinity, and cold [10–12]. These benefits are particularly evident in arid and semi-arid plants such as agaves, including autotrophy, biofilm formation, and water and nutrient retention [2, 13] together with protection against UV radiation, increasing the plant’s host resistance [14]. However, imbalances in the host-associated microbiome (*dysbiosis*) associated with management practices could cause plants to lose healthy functions such as digestion of complex polysaccharides, plant cell wall degrading enzymes, nitrogen fixation, and protection against soil pathogens, [15–17], low activity of bio-fertilization, bio-stimulation and biocontrol [18].

The *Agave* genus, which is influenced by both natural and artificial factors, provides a compelling example of these processes. Comprising around 210 species primarily distributed in arid and semiarid regions across Mexico and the southern United States [19–21], *Agave* species have been historically utilized for diverse purposes, including human and animal food, biofuel, fiber, medicine, and the production of fermented beverages and spirits [21]. In these species, domestication syndromes characterized by traits such as stem gigantism, higher carbohydrate concentration, reduced leaf dentition, and lower levels of saponins to facilitate harvesting have been identified [21]. The significant increase in *Agave* spirit production since the nineteenth century reflects recent management practices, which, along with the long-life cycle of *Agave*, accounts for the early stages of domestication seen in cultivars and landraces [21, 22]. However, the establishment of large monocultures, particularly when the *Agave* species involved can produce clones, results in a significant loss of genetic diversity, making these monocultures highly vulnerable to pathogens and pests. The loss of soil quality, high incidence of pests, and extensive use of agrochemicals, including herbicides, further contribute to environmental contamination and the reduction of associated microbial diversity and functions [2]. These microbial associations are key to promoting the health and growth of *Agave* plants. The relationships are maintained through the mutualistic interactions facilitated by mycorrhizae and other microorganisms, indicating that both the plants and their associated microbial communities are part of an interconnected system, coexisting through fungal and bacterial-mediated interactions. Therefore, one alternative for achieving sustainable mezcal production in arid and semi-arid regions is the rational use of microbial consortia adapted to these extreme habitats and their specific hosts [23, 24]. This approach leverages microbial biodiversity, incorporating it into integrated and sustainable production systems. This study evaluated the effects of agroecological management versus monoculture and wild populations on the microbial communities associated with *A. angustifolia*, the most widely used mezcal-producing species in Mexico, across three key production regions in the north, central, and southern parts of the country.

## Materials and Methods

### Sample Collection and Processing

**Fig. 1:**
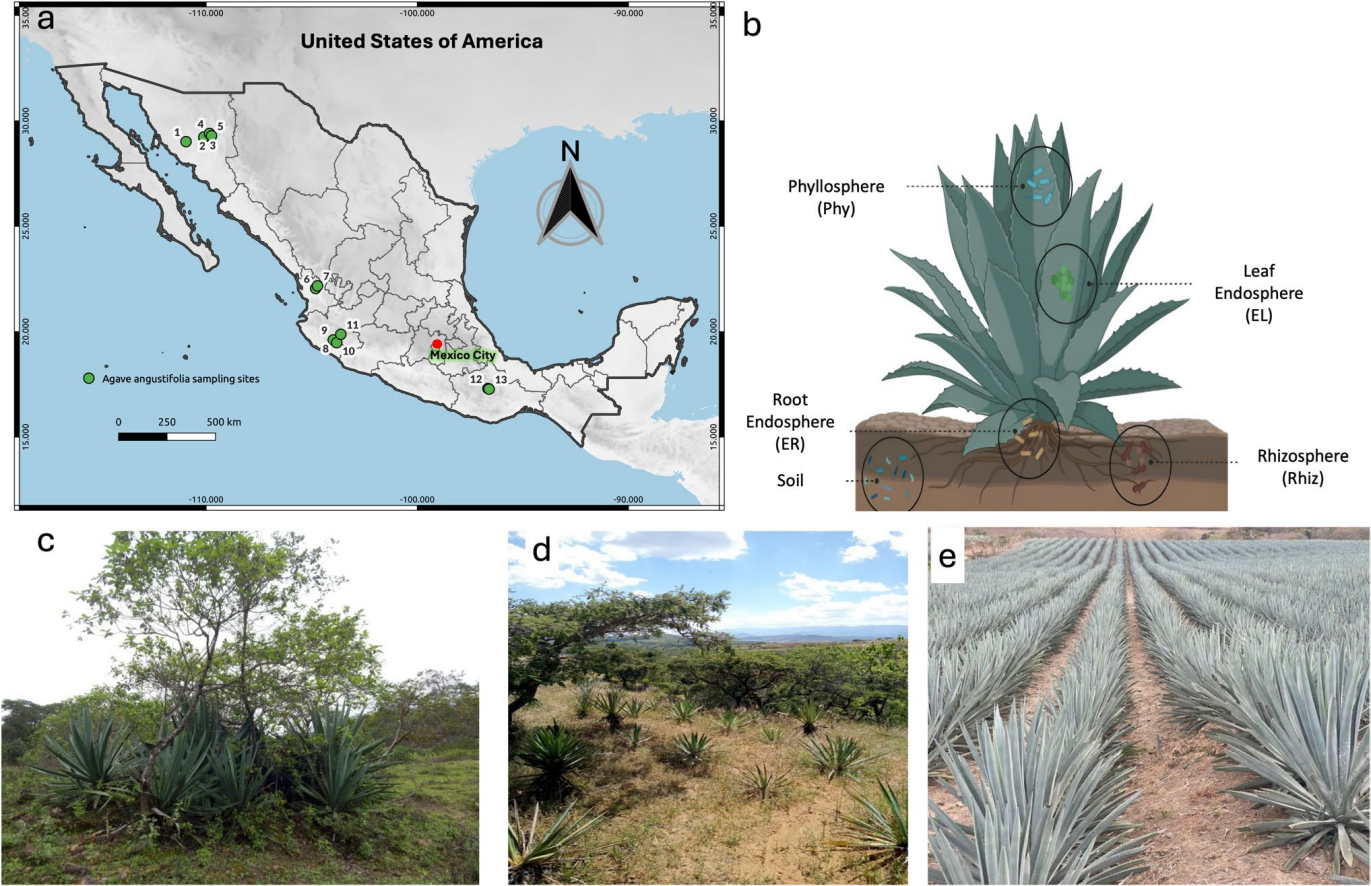
Experimental design for *A. angustifolia* microbiome analysis: **a** *A. angustifolia* sampling sites from across Mexico. Code numbers correspond to Table 1. **b** Samples analyzed for each plant individual: The images represent the different management statuses of *A. angustifolia* **c** wild, **d** traditional, and **e** conventional

*Agave angustifolia* Haw is the most widespread species of the genus in Mexico, occurring from northern Mexico in the states of Chihuahua and Sonora to Nicaragua in Central America. It inhabits a wide variety of plant communities, such as coastal dunes, low deciduous and sub-deciduous forests, xerophytic scrub, and pine-oak forests. Likewise, throughout its distribution, it is used in a variety of ways as a spirit drink, to obtain fiber, and as a medicinal plant [19, 21]. In 2022, we conducted a comprehensive collection of samples from 13 sites across the Mexican states of Sonora, Nayarit, Jalisco, and Oaxaca, encompassing most of the geographic distribution of *A. angustifoli*a. These sites were chosen to represent a spectrum of agricultural management practices. Specifically, the sites were categorized based on their management status into three distinct groups: wild, traditional, and conventional. This categorization is referred to as “status” throughout this document (see Fig. 1a; Table 1). Wild sites were natural populations untouched by human agricultural activity, providing a baseline for natural conditions. Traditional sites include age-old farming practices that typically involve low input and organic methods, silviculture plantations, often passed down through generations. Conventional sites used modern agricultural techniques, including chemical fertilizers, pesticides, and clonally generated monocultures, reflecting current industrial farming standards. Each collection site was classified according to the De Martonne aridity index (hereafter IAM) following Rahimi et al. [25]. The De Martonne aridity index is based on the aridity index *I* = *P*/ (*T* + 10), where *T* is the mean temperature (°C), and *P* is the mean annual precipitation in mm of each collection site [25]. The sampling sites were assigned to AR, arid; SAM, semiarid Mediterranean; SH, subhumid; HUM, humid conditions according to the IAM (see Table 1).

**Table 1:** Geographic location, status management, De Martonne Aridity Index (IAM) and bioclimatic variables of the collection sites of *Agave angustifolia* populations sampled across Mexico. Precipitation of wettest month (bio13), mean temperature of driest quarter (bio9), isothermality (bio3), mean diurnal range (bio2)

Population	State	Locality	Status	Latitude	Longitude	IAM	bio13	bio9	bio3	bio2
1	Sonora	Huepari	Wild	29.402	− 109.840	SAM	151.00	21.63	52.98	19.23
2	Sonora	San Pedro	Traditional	29.294	− 109.746	SAM	143.00	22.48	53.03	19.57
3	Sonora	Alamos	Conventional	29.233	− 110.107	SAM	168.00	19.70	56.55	19.79
4	Sonora	Mazatan	Wild	29.211	− 110.109	SAM	164.00	19.95	56.59	20.03
5	Sonora	Centro Ecológico	Wild	29.015	− 110.954	AR	84.00	21.55	55.49	18.70
6	Nayarit	Nayar3	Wild	22.172	− 104.727	HUM	278.00	21.45	64.93	16.17
7	Nayarit	Nayar2	Wild	22.048	− 104.816	HUM	318.00	26.18	65.26	15.47
8	Jalisco	Sayula	Wild	19.873	− 103.622	SH	200.00	18.35	63.74	15.81
9	Jalisco	Toliman	Wild	19.601	− 103.919	SH	133.00	23.85	70.12	18.25
10	Jalisco	Toliman	Traditional	19.597	− 103.917	SH	137.08	23.78	70.08	18.34
11	Jalisco	Tetepan	Traditional	19.47	− 103.815	HUM	165.00	22.85	69.74	16.46
12	Oaxaca	Huitzo	Conventional	17.300	− 96.652	SH	166.00	13.55	66.98	10.72
13	Oaxaca	Huitzo	Traditional	17.275	− 96.606	SH	164.00	14.42	66.04	11.43

Samples were collected from three healthy plants at each collection site. For each sampled individual, four sub-samples were collected, representing the endosphere (leaves and root endosphere), the episphere (phyllosphere and rhizosphere) and the bulk soil. Two leaves for the leaf endosphere and phyllosphere and root tissue for root endosphere and rhizosphere were collected. Rhizosphere (microbial communities within the first 2 mm from the roots) and phyllosphere (leaf surface microbial communities) samples were prepared by washing collected roots and leaves with sterilized epiphyte buffer (50 mM KH_2_PO_4_, 50 mM K_2_HPO_4_, 0.1% Triton X-100). For the root and leaf endosphere, sampled root and leaf tissues were surface sterilized with sterilization buffer (1:20 dilution of commercial bleach) for 15 min while mixing. After sterilization, root and leaf tissues were dissected into thin sections and placed into a 15% glycerol stock in 50 ml plastic tubes. Bulk soil was sampled by collecting topsoil (20 cm depth, ~ 50cm^3^) approximately 1 m from the individual sampled; the soil subsamples were pooled per site (Fig. 1b). All processed samples were stored at − 80 °C for further DNA extraction. The final data set depended on the quality of the DNA samples for each plant compartment. A total of 254 samples were analyzed (137 for 16S prokaryotes and 137 for ITS2 fungi). The characteristics of the samples for both 16S and ITS2 markers, including the location, type of sample, status, and IAM, are summarized in Table S1 .

### DNA Extraction and Sequencing

DNA extraction of root and leaf endosphere was performed using the Zymo Research Quick-DNA Plant/Seed Miniprep Kit; for phyllosphere and rhizosphere, we used the Zymo Research Microbiome Minikit; and for soil samples, the DNeasy PowerSoil Pro Kit (Qiagen). In all cases, the manufacturer’s instructions were followed. High-quality DNA was sent to AIM—Advanced Identification Methods GmbH (Leipzig, Germany) for sequencing services as follows: for ITS2 and 16S region amplification, we used the ITS29F/ITS24R and 515F/816R primer pairs, respectively [2]. For 16S amplification, peptide nucleic acid (PNA) clamps were used as reported by Lundberg et al., [26], which significantly may reduce chloroplast and mitochondrial contamination and increased prokaryotic reads for root endosphere and leaf endosphere. Paired end 2 × 300 bp sequencing was performed, in two independent runs for each amplicon, on an Illumina MiSeq instrument (Illumina Inc., San Diego, CA, USA). The sequences associated with this project are available in SRA Bio Project **PRJNA1168991.**

### Data Processing

Prokaryotic and fungal FASTQ reads were processed independently using the DADA2 pipeline [27], which characterizes microbiome composition through unique sequence variants, known as amplicon sequence variants (ASVs). In the R software, the 16S reads from each of two independent runs were first filtered and trimmed using DADA2 with the following parameters: Run_1: truncLen = c (220,180), maxN = 0, maxEE = c (2,6), truncQ = 2 and, Run_2: truncLen = c (250,180), maxN = 0, maxEE = c (2,6), truncQ = 2. For both ITS2 runs, we used with the following parameters: maxN = 0, maxEE = c (2,6), truncQ = 2. The filtered reads for each run for both 16S and ITS2 genes were then dereplicated and denoised using DADA2 default parameters. After removing chimeras, an ASV’s table of each run was constructed and then merged for each gene (i.e., using the “*mergeSequenceTables*” function from “*phyloseq*” R). Taxonomic assignments for the merged non-chimeric ASV’s table were performed using the Naïve Bayesian Classifier method [28], implemented in DADA2. For the 16S sequences, we used a training dataset formatted for DADA2 classifier based on the Silva v.138 reference database [29]. For the ITS2, the assignment was made against a classifier based on the UNITE ITS2 database (ver.10.0 [30]).

### Statistical Analysis

#### Taxa Filtering and Alpha Diversity Analysis

Results for ASV’s in 16S and ITS2 amplicon pipelines (DADA2) were imported into phyloseq objects (“*phyloseq*” package) for downstream bioinformatic analysis implemented in R (R project). For the 16S amplicons, the phyloseq object was trimmed of chloroplast and mitochondrial-like sequences, low prevalence, and non-taxonomic phyla.

Total sum scaling (TSS) normalization was used for alpha diversity measurements in both 16S and ITS2 markers. TSS uses the total read counts for each sample as size factors to scale the matrix counts. To normalize the count data, the ASV read counts are divided by the total number of reads in each sample to convert the counts to proportions, and the total number of ASVs (binned fragments) in the sample is used to adjust the abundance of each ASV [31]. Alpha diversity per sample (i.e., Shannon, Inverse Simpson, Richness, and Chao-1) was calculated using the “*microbiome*” package and compared among sample type/compartment, status, and IAM. Shared and unique ITS2 and 16S ASVs among sample type, IAM levels, and status were calculated and visualized with a Venn diagram using the “*ps_venn*” function of the “*MicEco*” package.

#### Correlation of Alpha Diversity with Bioclimatic Variation

To test the relationships between alpha diversity (i.e., Shannon diversity index) for both the whole microbiome and each compartment (i.e., leaf endosphere, root endosphere, phyllosphere, and rhizosphere) and the climatic variation, data for 19 bioclimatic raster layers available in WorldClim 2.1 [32] for current conditions (1970–2000) with 30 arcsecond resolution (available at: https://www.worldclim.org) were obtained for each collection site. A variance inflation factor, VIF [33], was applied to select the minimum redundant bioclimatic layers (variables with VIF < 10 were included). The resulting variables (i.e., bio13, bio9, bio3, bio2) after the VIF analysis were used to construct additive linear regression models against the Shannon diversity index using the entire dataset and for each compartment through the “*stats*” package in R. In addition, we constructed a fully reduced model using AIC-based stepwise regression (“*stepAIC*,” “*MASS*” packages) to determine the most parsimonious model explaining the relationships between alpha diversity (i.e., Shannon diversity) and bioclimatic variation. Microbial community composition analyses for key microbial players were plotted using the average relative abundance of each ASV within each taxonomic level for plant compartment, Status, and IAM communities using the “*MicrobiotaProcess*” library R software.

### Beta Diversity Analysis

Phylogenetically based unweighted *UniFrac* distances were calculated using the “*vegdist*” function of the “*vegan*” package for beta diversity analysis. Two-dimensional nonmetric multidimensional scaling (NMDS) was performed using the “*phyloseq*,” “*vegan*,” and “*ggplot2*” packages, including sample type, status, and IAM. Statistical significance of differences in beta diversity between plant compartment, status, and IAM was tested using permutational multivariate analysis of variance (PERMANOVA). PERMANOVA’s were also performed per compartment to test for differences in status and IAM levels (*p* < 0.05). PERMANOVA’s were performed using the “*adonis*” function of the R “*vegan*” package. Additionally, the correlation between geographic distances and bioclimatic variables (i.e., bio13, bio9, bio3, bio2) and prokaryotic and fungal community composition (i.e., Unweighted UniFrac distances sample/site means) was analyzed by Mantel tests (permutations = 9999), for the whole community and for each compartment.

### Differential Analysis of Microbiome Compositions with Bias Correction

Differential abundance (DA) analysis was performed using microbiome composition analysis with bias correction (“*ANCOM-BC*” package) at the genus level for each compartment (p and false discovery rate (FDR) < 0.05). Significance comparison between management statuses was performed using log fold changes (LFC); only leaf endosphere were excluded due the low number of ASV’s to compare among statuses (Table S3).

## Results

For the 16S gene, a total of 3,654,741 high quality reads were retained, clustering into 8,214 ASVs, after filtering and removal of mitochondrial and chloroplast-like sequences. On the other hand, the ITS2 data yielded a total of 2,073,457 high quality reads retained after filtering, clustered into 7,459 ASVs. The Venn diagram showed a core of 100 ASVs shared among the type compartments for 16S data, whereas the endosphere and episphere harbored 1,291 and 4,837 ASVs, respectively (Fig. S1), while the soil harbored 1,132 exclusive ASVs. By Status, a core of 457 ASVs was found, while wild and traditional Status harbored 3,973 and 2,013 ASVs, respectively (Fig. S1), while conventional Status harbored 964 exclusive ASVs. The IAM classification showed a core of 164 ASVs, while Semiarid Mediterranean (SAM) and Humid (HUM) harbored the highest number of exclusive ASVs (2,630 and 190, respectively; Fig. S1). For ITS2, no ASVs were shared among compartments (i.e., endosphere, episphere, and soil, Fig. S2), the highest number of exclusive ASVs were found in the endosphere (3,585) and episphere (3,256), which share less than 1% of ASVs, and soil (580), which shares ASVs only with episphere samples. By status, a core of only 6 ASVs was found, while wild and traditional status harbored 3,657and 2,458 ASVs, respectively (Fig. S2), and conventional status harbored 1,240 exclusive ASVs. The IAM classification showed no core ASVs and less than 1% shared between IAM levels, while SH and SAM harbored the highest number of exclusive ASVs (2,730 and 2,374 ASVs, respectively, Fig. S2).

### Alpha Diversity

**Fig. 2:**
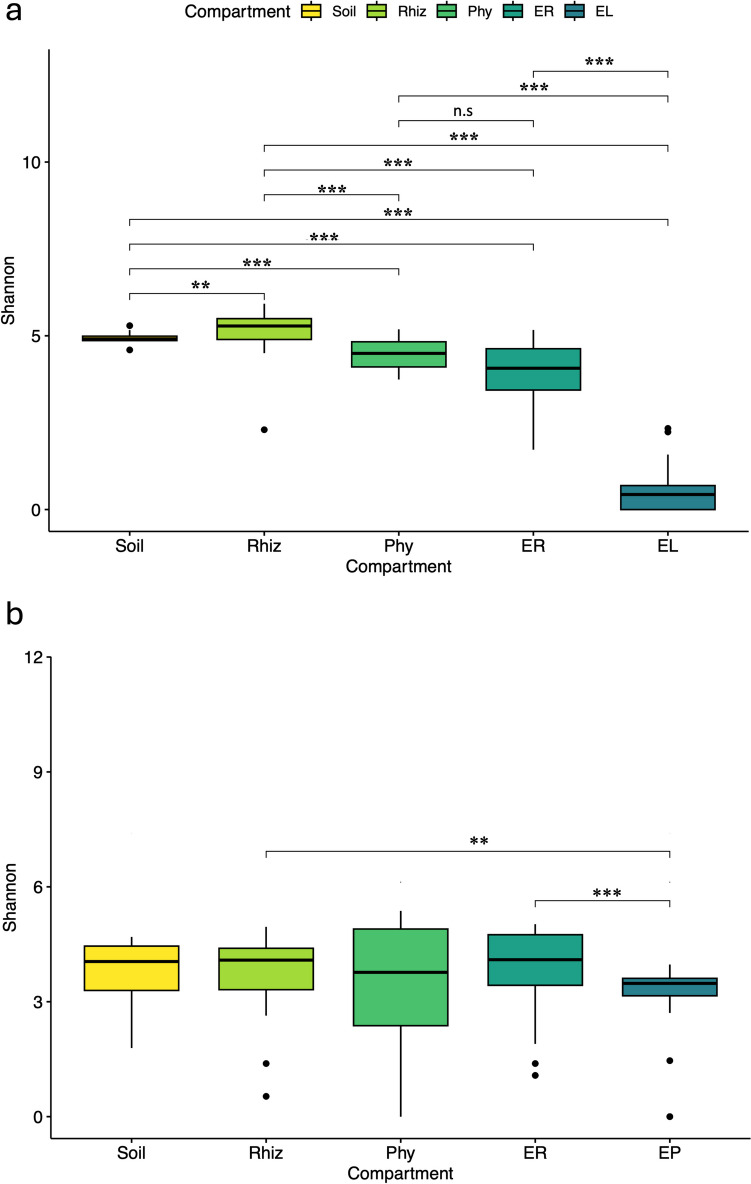
Shannon diversity index among different plant compartments and soil for **a** prokaryotic and **b** fungal communities (**b** and **c**) associated with *A. angustifolia*. Significant differences (*X*^*2*^* Kruskal–Wallis*): ***p = 0; **p < 0.001. Only significant differences are shown

**Fig. 3:**
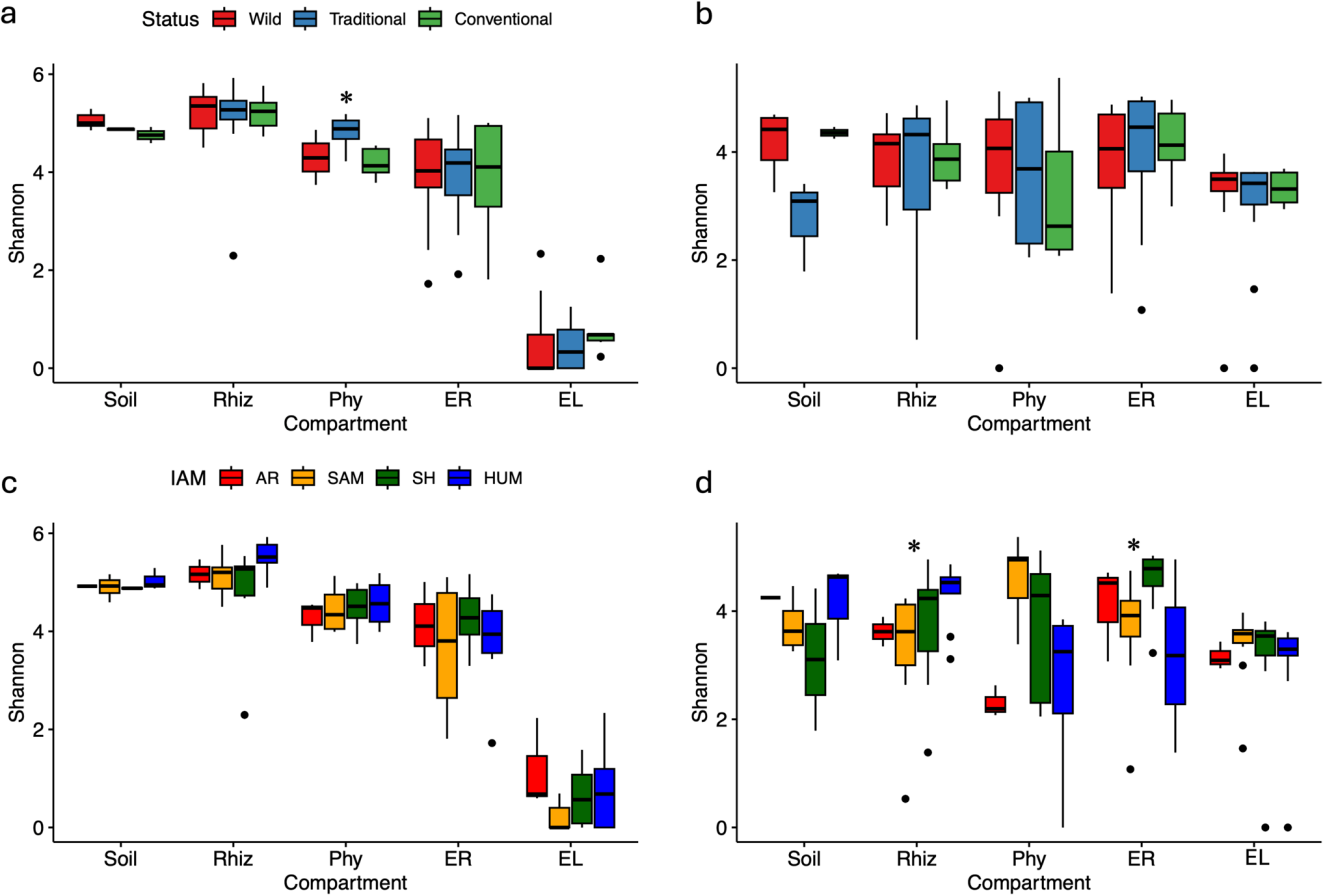
Shannon diversity index in the prokaryotic (**a** and **c**) and fungal communities (**b** and **d**) associated with the management status and De Martonne aridity index (IAM) for *Agave angustifolia* populations from across Mexico, respectively. *Significant differences (*X*^*2*^* Kruskal–Wallis*) at compartment level for each status and IAM level are shown. AR, arid; SAM, semiarid Mediterranean; SH, subhumid; HUM, humid

**Fig. 4:**
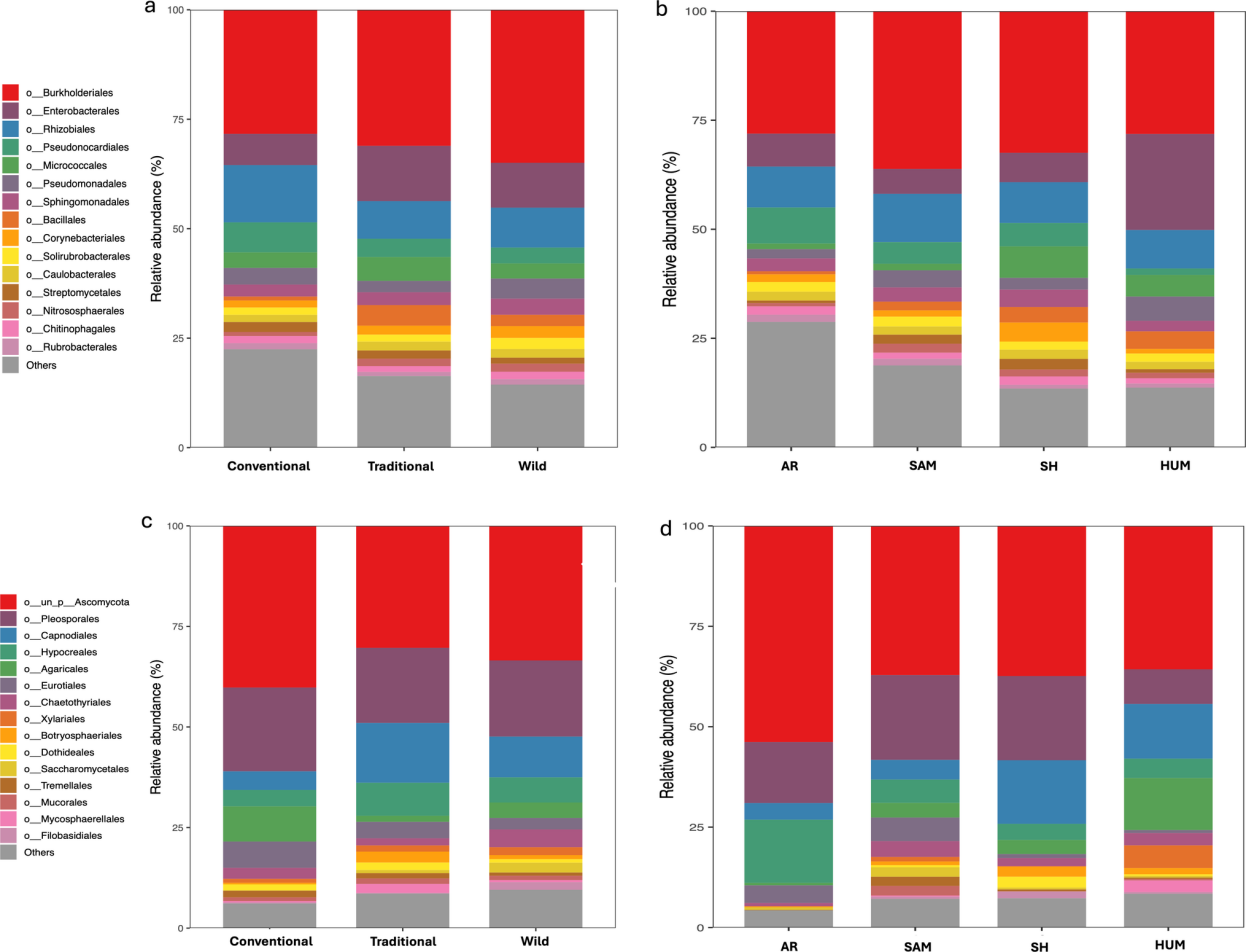
Order-level relative abundance plots of global microbiome for prokaryotic taxa for Status (**a**) and IAM (**b**) in samples of *A. angustifolia* from across Mexico. Order-level relative abundance plots of fungal taxa for status (**c**) and IAM (**d**) in samples of *A. angustifolia* from across Mexico

A marked increase in alpha diversity for the 16S gene (i.e., Shannon, observed, and Chao1 indices) was found from the endosphere to the rhizosphere and soil (Fig. 2a; Table S2). For ITS2, similar alpha diversity values were found among compartments, with the leaf endosphere exhibiting the lowest alpha diversity values, while the episphere compartments showed the highest values (Fig. 2b). Overall, alpha diversity showed no significant differences between Status and IAM for the entire dataset for both 16S and ITS2 markers (Fig. 3). However, at the compartment level for the 16S gene, significant differences between Status and IAM were found for phyllosphere Shannon diversity (*X*^2^ = 6.55, *p* = 0.03; Fig. 3a and b). For ITS2 markers, significant differences were observed at IAM for the root endosphere (*X*^2^ = 10.81, *p* = 0.01; Fig. 3d) and the rhizosphere (*X*^2^ = 9.25, *p* = 0.02; Fig. 4d).

### Relative Abundance Taxa

Among the different samples, 32 distinct prokaryotic phyla were identified in *Agave angustifolia* samples. Proteobacteria, Actinobacteria, and Firmicutes dominated, comprising up to 80% of the microbial community across different Status and IAM (Fig. S3 and S4). These phyla were especially prevalent in endosphere samples. In contrast, episphere and soil samples also exhibited high abundances of Bacteroidota, Crenarchaeota, and Chloroflexi (Fig. S10 and S11). At the order level, Burkholderiales, Enterobacterales, Rhizobiales and Pseudonocardiales dominated with up to 50% abundance between Status and IAM, while among compartments, Burkholderiales showed high prevalence in leaf compartments (leaf endosphere and phyllosphere), while Enterobacterales, Burkholderiales, Rhizobiales, Pseudonocardiales, Micrococcales, Pseudomonadales dominated in high relative abundance for root compartments (root endosphere and rhizosphere) and soil (Fig. S5 and S6). At the phyllosphere, a marked reduction at conventional status was found for Rhizobiales (*X*^2^ = 5.46, *p* = 0.04) with a marked increase in Lactobacillales (*X*^2^ = 6.28, *p* = 0.04); for the rhizosphere, an increase in cultivated status was found for Rhizobiales (*X*^2^ = 7.152, *p* = 0.02) while Bacillales (*X*^2^ = 5.49, *p* = 0.04; Fig. S5), showed a substantial reduction. For IAM, at rhizosphere an increase in humid sites for Micrococcales (*X*^2^ = 15.99, *p* = 0.001), Enterobacterales (*X*^2^ = 14.88, *p* = 0.001), Bacillales (*X*^2^ = 9.70, *p* = 0.02) was observed. For root endosphere, Enterobacterales (*X*^2^ = 10.70, *p* = 0.01) increased in aridity, while Pseudomonales increase slightly in humid and semi-arid sites (*X*^2^ = 9.71, *p* = 0.02; Fig. S6).

For ITS2 data, 11 different fungal phyla were found across *A. angustifolia* samples, with Ascomycota and Basidiomycota dominating with up to 90% relative abundance across Status and IAM (Fig. S11). Among the Status and IAM samples, the order Ascomycota Incertae sedis dominated, comprising up to 50%, followed by Pleosporales, Capnodiales, Eurotiales, and Hypocreales, which together accounted for up to 30% (Fig. S12). Across different compartments, from leaf endosphere to soil, the relative abundance of Ascomycota Incertae sedis decreased, while Pleosporales, Capnodiales, Eurotiales, Hypocreales, and Agaricales increased in relative abundance (Fig. S12).

At compartment level, only for soil the orders Pleosporales (*X*^2^ = 6.23, *p* = 0.04) and Agaricales (*X*^2^ = 5.87, *p* = 0.04) showed an increase in cultivated populations (Fig. S8). Otherwise, for IAM, Pleosporales (*X*^2^ = 9.41, *p* = 0.02) and Xylariales (*X*^2^ = 10.31, *p* = 0.01), displayed higher prevalence in semiarid zones while decreasing in humid and sub-humid sites. For root endosphere, an increase in prevalence for Pleosporales (*X*^2^ = 12.54, *p* = 0.005) and Hypocreales was found in subhumid sites (Fig. S9).

### Correlation of Alpha Diversity and Bioclimatic Variables

**Fig. 5:**
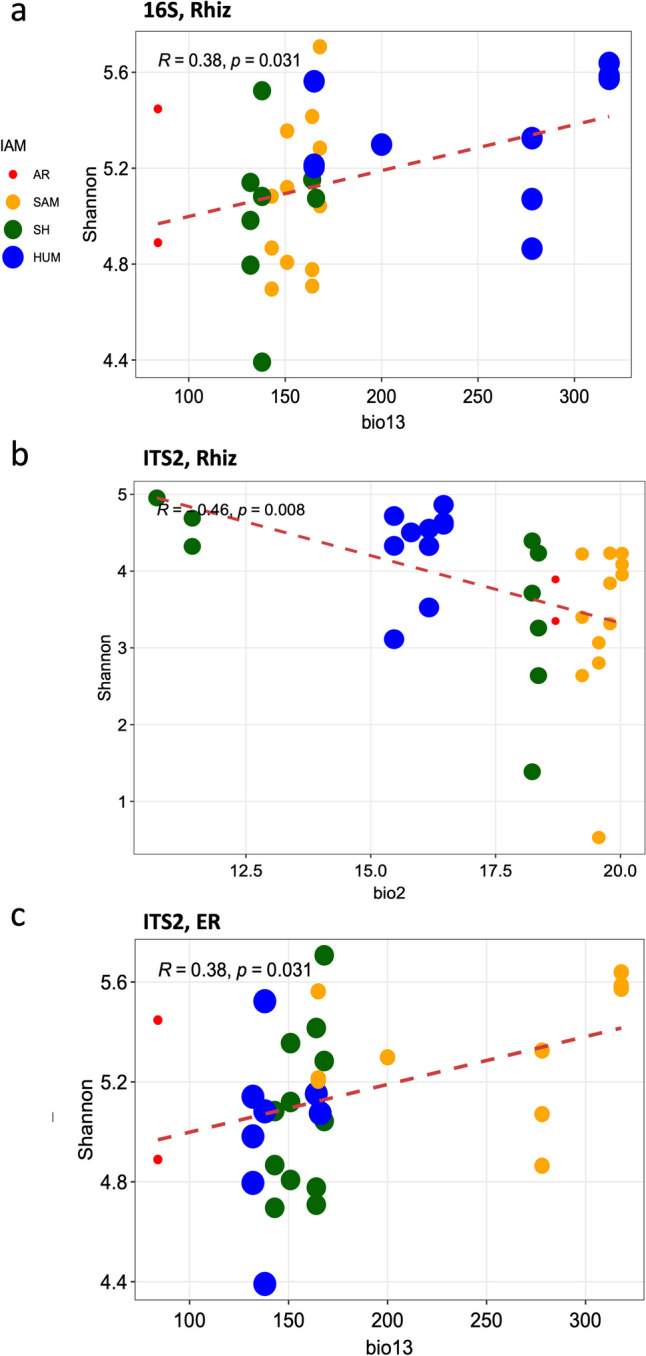
Linear regression among compartment prokaryotic and fungal Shannon diversity and bioclimatic variables of the collection sites. **a** Prokaryotic rhizosphere Shannon diversity index and the Precipitation of Wettest Month (bio13), **b** fungal rhizosphere Shannon diversity and mean diurnal range (bio2), **c** fungal root endosphere Shannon diversity and Precipitation of Wettest Month (bio13)

The results of the linear model including the bioclimatic variables resulting from the VIF and the Shannon diversity index for the ITS2, showed negative correlations among the root endosphere Shannon diversity index and the bio13 (Precipitation of Wettest Month; AIC = −5.27; *R* = − 0.48, *p* = 0.003; Fig. 5a), as well as among the rhizosphere Shannon diversity index and the bio2 (mean diurnal range; AIC = −6.08; *R* = − 0.46, *p* = 0.008; Fig. 5b). Otherwise, 16S gene showed only a positive correlation between the rhizosphere Shannon diversity index and the bio13 (Precipitation of Wettest Month; AIC = −72.13; *R* = 0.38, *p* = 0.031, Fig. 5c).

### Beta Diversity Patterns Among Management Statuses and IAM

**Fig. 6:**
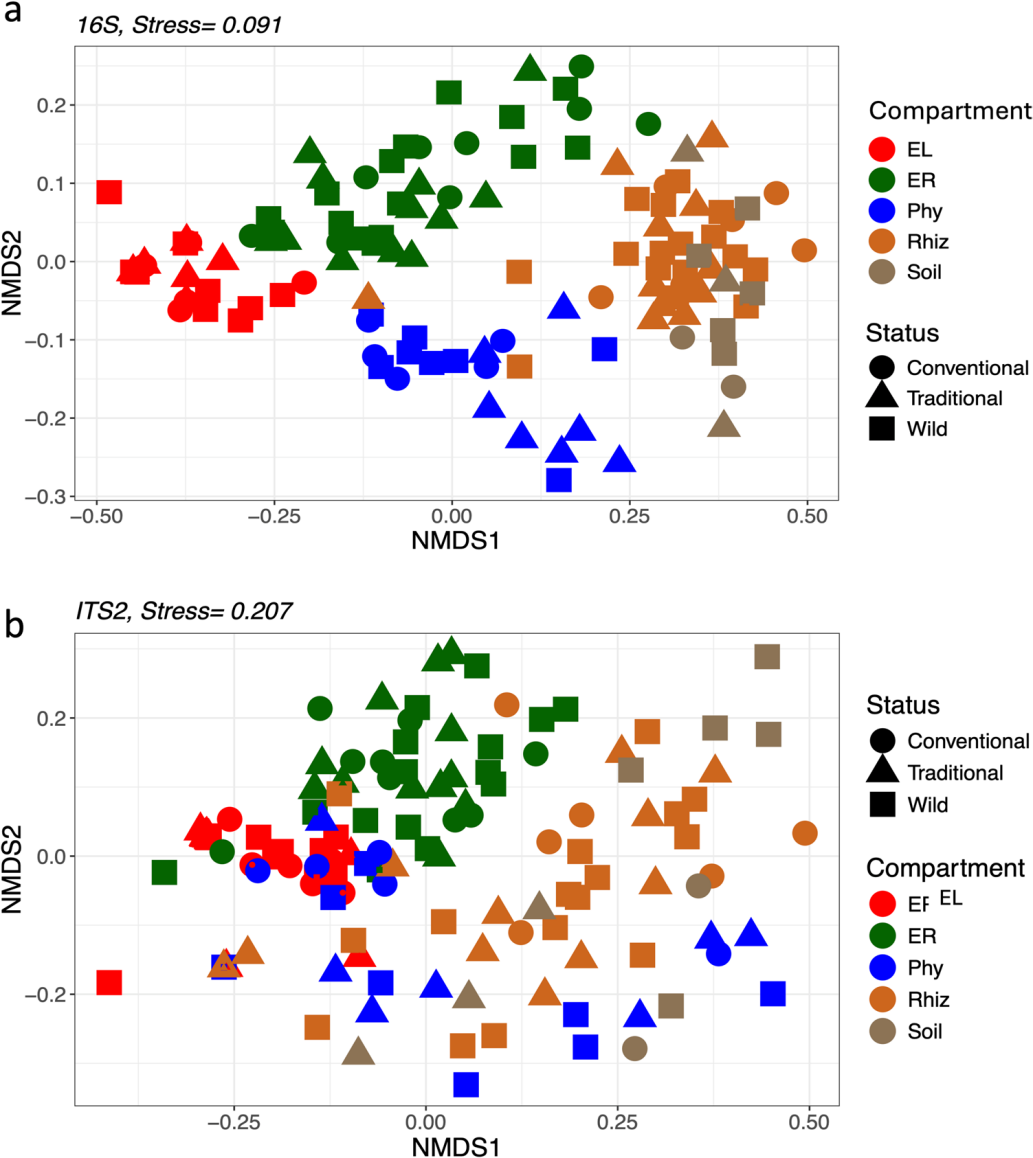
Nonmetric multidimensional scaling (NMDS) plots for UniFrac distances of prokaryotic and fungal communities from *A. angustifolia* populations under different management status from across Mexico. **a** Prokaryotic communities from the different compartments according to the management status, **b** fungal communities from the different compartments according to the management status

**Fig. 7:**
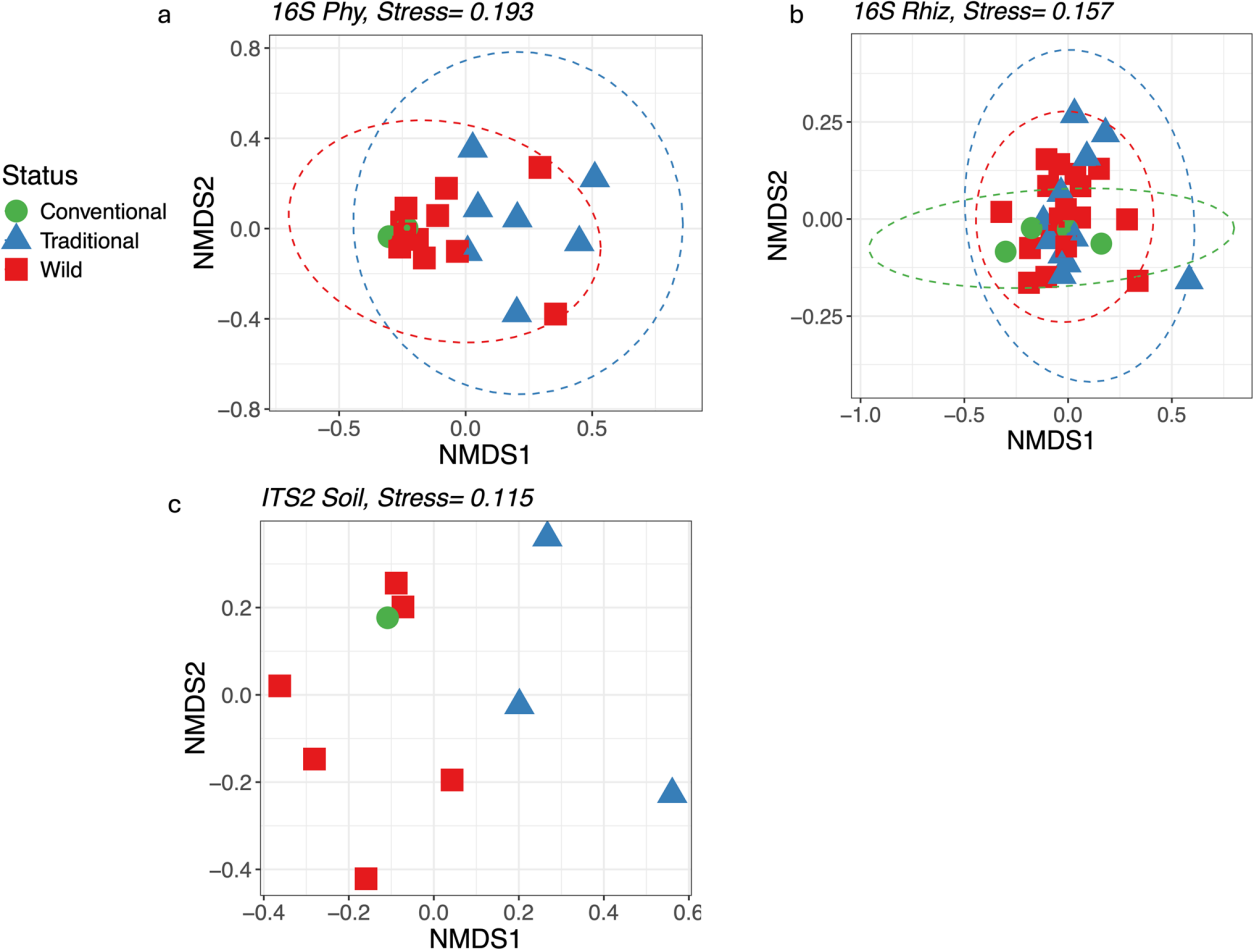
Nonmetric multidimensional scaling (NMDS) plots for UniFrac distances of microbial communities at compartment level from *A. angustifolia* populations under different management status: prokaryotic communities: **a** phyllosphere; **b** rhizosphere; fungal communities: **c** soil

In agreement with the PERMANOVA results (Table 2), NMDS analysis showed a clear separation between the microbial communities of the plant compartments for both 16S and ITS2 markers (Fig. 6a and b). However, PERMANOVA analysis revealed no significant differences when evaluating the effect of status on beta diversity patterns for the global microbiome using the 16S marker (*R*^2^ = 0.013, *p* = 0.38; see Fig. 6a) and ITS2 (*R*^2^ = 0.015, *p* = 0.08; see Fig. 6b). Nevertheless, the differences in major taxa orders between management statuses (i.e., Fig. 3a and c) and the total number of exclusive ASVs found for each management status reveal underlying differences between microbiomes from different management statuses (Fig. S1 and S2). For example, significant effects of management status were found for sample type (i.e., episphere; *R*^2^ = 0.044, *p* = 0.04; Table 2), for prokaryotic communities. In line, at the compartment level, the effect of management status on prokaryotic community composition showed significant differences for phyllosphere (*R*^2^ = 0.144; *p* = 0.01) and rhizosphere (*R*^2^ = 1.188; *p* = 0.04), consistent with the NMDS grouping by compartment (Fig. 7a, b). On the other hand, significant differences for the ITS2 marker were found at the compartment level (Table 2), where the effect of management status was significant for soil, demonstrating that agricultural practices significantly shape the microbiome at this scale, as shown by NMDS analyses for each compartment (Fig. 7c; Table S3).

**Table 2:** PERMANOVA analysis of the microbial communities associated with *A. angustifolia*, for the global microbiome, episphere (Rhiz, Phy) and endosphere compartments (RE, LE) considering compartment, management status and IAM. *AR*, arid; *SAM*, semiarid Mediterranean; *SH*, subhumid; *HUM*, humid

16S					ITS2				
Global		*F*	*R*^2^	*p*	Global		*F*	*R*^2^	*p*
Status	Compartment_2,122_	13.576	0.175	**0.0001**	Status	Compartment _2, 125_	8.174	0.200	**0.0001**
	Status_2,122_	1.014	0.013	0.3877		Status _2,125_	1.301	0.015	0.0805
	Compartment:Status_4, 122_	0.986	0.025	0.4620		Compartment:Status _4, 125_	1.139	0.055	0.0900
IAM	Compartment_2, 119_	13.747	0.175	**0.0001**	IAM	Compartment _2, 119_	7.085	0.096	**0.0001**
	IAM_3, 119_	1.383	0.026	0.0617		IAM _3, 119_	1.560	0.032	**0.0079**
	IAM:Compartment_6, 119_	1.146	0.044	0.1528		IAM:Compartment _6, 119_	1.189	0.048	0.0629
**Episphere**					**Episphere**				
Status	Status_2, 54_	1.196	0.039	0.137	Status	Status _2, 47_	1.177	0.043	0.1141
	Compartment_2, 54_	9.166	0.149	**0.0001**		Compartment _1, 47_	2.562	0.047	**0.0002**
	Status:Compartment_4, 54_	1.367	0.044	0.049		Status:Compartment _2,47_	1.141	0.042	0.1382
IAM					IAM				
	IAM_3,51_	1.652	0.066	**0.0022**		IAM_3, 45_	1.597	0.085	**0.0001**
	Compartment_2, 51_	5.810	0.156	**0.0001**		Compartment_1, 45_	2.634	0.047	**0.0001**
	IAM:Compartment_6, 51_	1.165	0.094	0.0674		IAM:Compartment_3, 45_	1.386	0.073	**0.0020**
**Endosphere**					**Endosphere**				
Status	Status_2, 62_	1.101	0.022	0.298	Status	Status_2, 62_	1.142	0.026	0.2302
	Compartment_1,62_	29.972	0.312	**0.0001**		Compartment_1,62_	18.25	0.207	**0.0001**
	Status:Compartment_2, 62_	0.830	0.0173	0.617		Status:Compartment_2, 62_	1.120	0.025	0.2577
IAM					IAM				
	IAM_3, 60_	1.801	0.054	**0.0216**		IAM_3, 60_	1.430	0.047	0.0500
	Compartment_1, 60_	29.496	0.297	**0.0001**		Compartment_1, 60_	18.956	0.082	**0.0001**
	IAM:Compartment_3, 60_	1.448	0.044	0.0824		IAM:Compartment_3, 60_	1.595	0.053	**0.0200**

In turn, IAM induced significant differences in prokaryotic communities for the episphere (*R*^2^ = 0.066, *p* = 0.002) and endosphere compartments (*R*^2^ = 0.054, *p* = 0.02; Table 2). At root compartments, IAM significantly influenced differences in prokaryotic communities (i.e., rhizosphere and root endosphere) and soil along the sampling sites of *A. angustifolia* (Table S3; Fig. S2 and S13). In addition, for the ITS2 marker, significant differences were found between IAM levels (*R*^2^ = 0.031, *p* = 0.007) for global microbiome composition. Also, PERMANOVA revealed significant differences for episphere (IAM: *R*^2^ = 0.084, *p* = 0.0001; IAM: compartment: *R*^2^ = 0.073, *p* = 0.002; Table 2) and endosphere samples (*R*^2^ = 0.052, *p* = 0.02). Interestingly, IAM differences among sampling sites showed that significant differences were found in all plant compartments (i.e., leaf endosphere, phyllosphere, root endosphere, rhizosphere), demonstrating that environmental variation is a determinant shaping both fungal and prokaryotic at compartment level microbial communities associated with *A. angustifolia* (Table S4; Fig. S14).

### Correlation of Beta Diversity with Geographic and Bioclimatic Variation

The mantel test among communities’ differences and geographic and bioclimatic variables, showed no differences for prokaryotic communities among unweighted UniFrac distances and geographic and bioclimatic variables. However, for ITS2 marker, positive correlations were found among unweighted UniFrac distances and geographic distances (*R* = 0.395, *p* = 0.001), and mean diurnal range (bio2) (*R* = 0.133; *p* = 0.002).

### Differential Analysis of Microbiome Compositions with Bias Correction

**Fig. 8:**
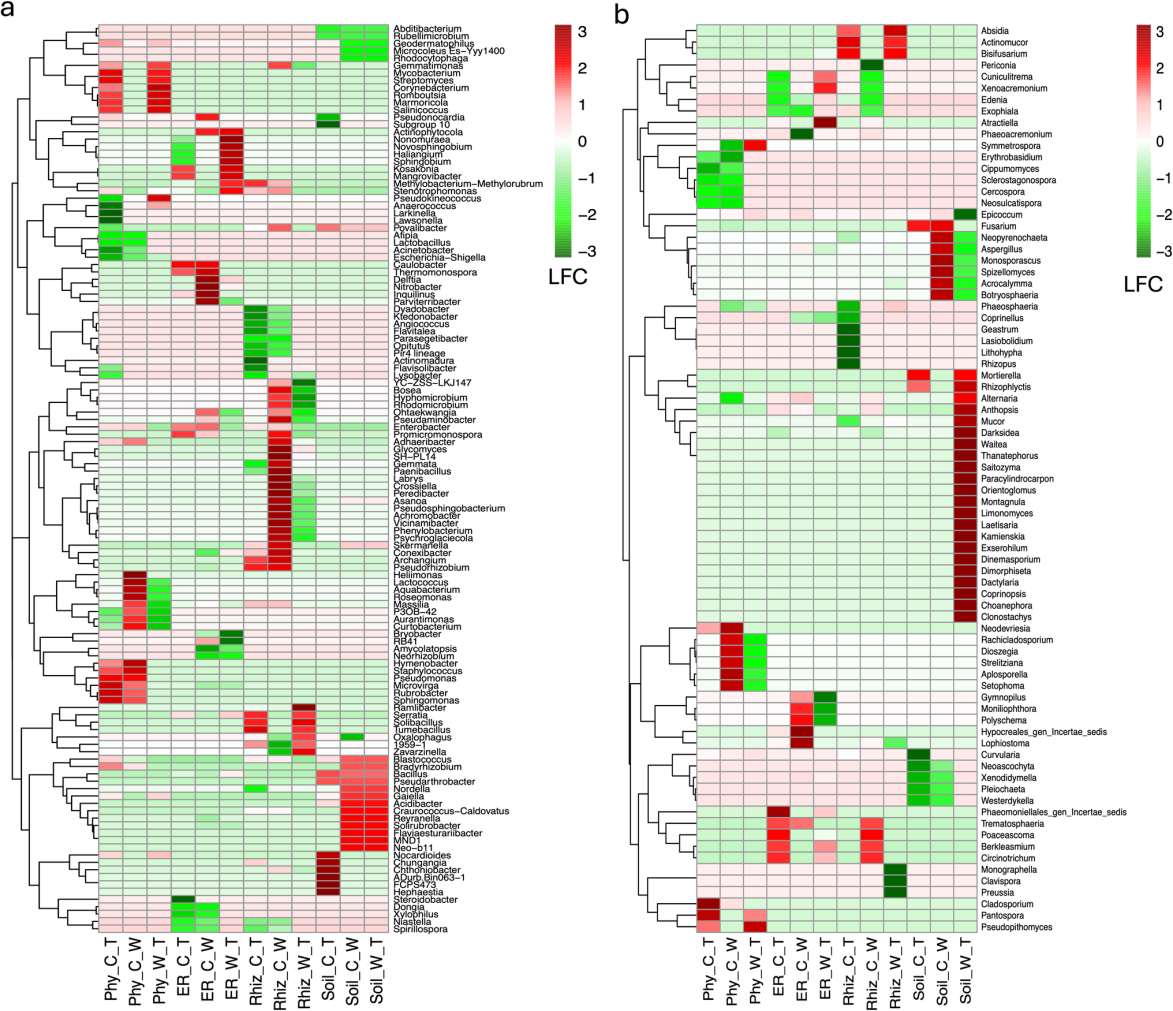
ANCOM-BC differential abundance analysis to determine significant log fold changes (LFC) differences (FDR < 0.05) for **a** prokaryotic and **b** fungal genus at compartment level comparing different management statuses: Phy, phyllosphere; ER, root endosphere, Rhiz, rhizosphere; soil, soil. C, conventional; T, traditional; W, wild. The letter for each code indicates the statuses being compared. The first letter of each code represents the positive LFC

The ANCOM-BC analysis identified bacterial and fungal taxa whose abundance was associated with different Status across various compartments. A total of 123 prokaryotic taxa were found for all interactions evaluated (Fig. 8a). For phyllosphere and soil in cultivated agaves, differential abundance for *Pantoea* (Proteobacteria) and *Pseudomonas* (Proteobacteria) (Fig. 6; Table S4) were observed. It also highlighted the differential abundance of Bacteroidetes genera in the root endosphere and rhizosphere of wild populations such as *Ohtaekwangia*, *Adhaeribacter*, and *Parasegetibacter*, *Dyadobacter*, and *Flavisolibacter* (Fig. 6; Table 3). In the cultivated rhizosphere and soil, the differential abundance of Firmicutes genera *Paenibacillus*, *Gaiella*, *Bacillus*, and *Tumebacillus* (Fig. 6; Table 3) was observed.

For fungi, an increase in differential abundance for the Ascomycota *Fusarium* and *Aspergillus*, in soils and root endosphere from cultivated populations compared to wild and traditional management (Fig. 8b). *Bisifusarium* was found in high prevalence on root endophytes and rhizosphere in both wild and traditional samples, and Glomeromycota taxa, such as *Orientoglomus* and *Kamienskia*, showed differential abundance in soils and root compartments from wild populations compared to cultivated ones (Fig. 8b; Table S5).

## Discussion

### Sample Type Is the Main Factor Shaping the Diversity and Composition of Prokaryotic and Fungal Microbiomes in *A. angustifolia*

In our results, compartment is the primary factor shaping plant–microbe interactions, impacting diversity and composition more significantly than other factors such as status and IAM, in line with previous reports on agaves and cacti from arid and semiarid areas [2–4].This appear to strongly stabilize host-symbiont cooperation by isolating symbionts to reward beneficial interactions and prevent uncooperative ones [2–4, 34]. For prokaryotes, in leaf compartments a dominance of Burkholderiales, Rhizobiales, and Lactobacillales indicates diazotrophic functions, CO oxidation, nutrient availability and tolerance to drought, salinity and cold [10–12], as well as light sensing in the phyllosphere (i.e., Lactobacillales in *A. tequilana)* [13]. In contrast, for *Agaves* and cacti from warm deserts, *Cyanobacteria* dominates in phyllosphere, associated with autotrophy, biofilm formation, and water and nutrient retention [2, 13, 35, 36]. Similar alpha diversity levels were observed for the phyllosphere and rhizosphere, but rhizosphere showed a higher diversity than the surrounding soil, suggesting that root interactions improve conditions by increasing microbial richness, shaping community composition and diversity [37, 38].

### Environmental Conditions Directly Affect the Diversity and Composition of Prokaryotic and Fungal Communities Along the Distribution of *A. angustifolia*

Consistent with this, we found an increase in alpha diversity in the rhizosphere that was directly associated with higher precipitation at the sampled sites (Fig. 5a). At wetter sites, the rhizosphere and root endosphere showed an increase in Micrococcales (e.g., *Pseudarthrobacter* spp.), Enterobacterales (e.g., *Enterobacter*, *Serratia*), and Bacillales (e.g., *Bacillus*, *Paenibacillus*), associated with plant growth promotion, antibacterial activity, nitrogen fixation, phosphorus solubilization, antibiotic and chitinase production, and biological control [39–41]. Pseudomonadales (i.e., *Pseudomonas*) are known as plant growth enhancers and tolerance to heatwaves, floods, and salinity [42], and were enriched in the root endosphere. Water availability and microhabitat conditions directly affect the microbiome in *A. angustifolia* [38], while geographic distance does not impact the prokaryotic communities.

Otherwise, variations in bioclimate were the key factors shaping diversity and composition in the fungal microbiome or mycobiome, consistent with previous studies suggesting that biogeography influence fungal communities more than other microbial communities [43]. An increase in alpha diversity was found at root compartments in drier and hotter sites (Fig. 5b and c), particularly dominated by Pleosporales and Eurotiales. Pleosporales have protective spores against UV and dryness, enhancing plant host resilience, while Eurotiales in *O. ficus-indica* aid in phosphate solubilization as saprotrophs [14]. These traits could assist plant hosts in coping with drought and desiccation under arid conditions.

Fungal endemism may result from specific environmental conditions in different habitats, with dispersal limitation likely being the main cause of differences in fungal communities [44]. Previous studies on plants such as grasses, *Opuntia* and *Agave* have also found correlations between fungal community differences and geographic distance along environmental gradients [2, 3, 45]. Our results from the Mantel test demonstrated a clear influence of geographic distance and temperature patterns on global fungal communities, like findings in *O. ficus-indica*, where these factors significantly affected composition but not alpha diversity levels [3]. This suggests that spatial distance and environmental differences play a significant role in shaping fungal microbial communities, as seen in studies on *A. tequilana* and wild agaves from North American arid zones [2].

### Management Status Influences the Diversity and Composition of the Microbiome at the Compartment Level in *A. angustifolia*

Although a decrease in microbial richness is not often found in cultivated plants compared to their wild relatives [46], a slight increase in prokaryotic alpha diversity was found for the phyllosphere under traditional management (Fig. 3). In managed crops, changes in microbiome diversity and composition are often associated with morphological and physiological changes in managed genotypes, but most reported for root compartments [6, 7, 9, 47]. In *A. tequilana*, a drastic reduction in phyllosphere alpha diversity compared to wild species was observed due to the dominance of a few prokaryotic families [2]. In our results, a slight reduction in conventional management was only found for Rhizobiales and Lactobacillales (Fig. S5). In *A. tequilana* monocultures, only the *Azul* variety is utilized for tequila production and is promoted via clones, disregarding the presence of 10 other varieties [20, 48]. This leads to lower genetic diversity compared to *A. angustifolia* [22, 48]. Instead, traditional management of *A. angustifolia* involves obtaining seedlings from vegetative propagules and seeds from different sources [22], as shown for populations used for Bacanora spirit production in Sonora, with an incipient history of cultivation and management, for which low genetic structure and high genetic diversity have been found [49]. Overall, these conditions can alter morphological and physiological variation and consequently affect microbial diversity. Similar findings were observed in the rhizosphere of maize [50, 51], potatoes [52], and barley [53], where alpha and beta diversity levels were directly associated with the analyzed genotypes. Moreover, in *Agave*, clonal propagation may influence microbial communities when passed down to offshoots, affecting microbial richness based on management practices [2].

While no differences were found in the global microbiome composition among management, differences at phyllosphere and rhizosphere for prokaryotes soil for fungi were found (Fig. 7), exhibiting the effect of management practices at the compartment level. Plant roots in species under selection showed an increase in Actinobacteria and Proteobacteria phyla, while Bacteroidetes decreased compared to their wild relatives [7, 47, 54, 55]. A decrease in Bacteroidetes prevalence was observed in the phyllosphere of cultivated populations. However, there were differential abundances of Bacteroidetes genera in the rhizosphere of wild populations, including *Ohtaekwangia*, *Adhaeribacter*, and *Parasegetibacter*, which are associated with plant growth promotion, phosphomonoesterase activities, phosphate and potassium dissolution, and nitrogen fixation [56–58]. Also, functions as synthesis of polysaccharide modifying enzymes, plant cell wall degrading enzymes, nitrogen fixation and soil pathogen protection as *Chitinophaga* and *Dyadobacter* [15–17] and *Flavisolibacter* which colonizes roots effectively, can contribute to biofertilization, biostimulation, and biocontrol activities, helping to limit the growth of *Fusarium oxysporum*. Interestingly, in the episphere of *A. tequilana*, a depletion of genes involved in carbon metabolism, secondary metabolite synthesis, and xenobiotic degradation was found, associated with the loss of microbes involved in these functions [13]. The functional impacts of changes in bacterial community composition due to domestication are not well understood and could be linked to compartment architecture. Bacteroidetes, known for digesting complex polysaccharides in wild plants and promoting plant growth in harsh environments, might play a role in this context [6, 47]. Also, in the human and mice gut microbiome, abundance of Bacteroidetes has been associated with low carbohydrate diets [18]. This association may lead to healthier evolution for plants, as shown for animals, and whether its increase in abundance on the roots of wild relatives is a signature of coevolution remains speculative.

Otherwise, at the cultivated rhizosphere differential abundance of bacterial general related with nitrogen metabolism as *Paenibacillus* and *Gaiella*, production of IAA and L-tryptophan and growth promoters as *Nocardioides*, *Bacillus*, and *Tumebacillus*, previously reported to be recruited at root cucumber some help with N fixation [41]. Also, markedly differential abundance of *Pseudomonas,* was found in cultivated phyllosphere which previously was isolated from *A. palmeri* and has been recognized as growth promoter and widely used as bio stimulant [59]. A differential prevalence of N-fixing bacteria in cultivated agaves could be due to the regular application of nitrogen fertilizer inputs, which are identified to promote intense bacterial competencies as well as to increase the complexity of interaction networks and the depletion of ammonia-oxidizing archaea [60]. On the other hand, in the phyllosphere and soil in cultivated agaves differential abundance for *Pantoea*, frequently associated with the “*soft rot disease*,” and in *A. tequilana* for which represents substantial annual losses [2] and reported for *A. angustifolia* [61, 62]. In terms of fungi, surprisingly, an increase in differential abundance for the Ascomycota *Fusarium* and *Aspergillus*, in soils from cultivated populations in comparison to wild and traditional managements was found (Fig. 8b). In *A. tequilana*, the soil-borne pathogens *F. xysporum* and *F. solani* have been identified as principal causing agent of the “*agave vascular wilt*” [63]. Otherwise, *Aspergillus* a saprotroph related with phosphate solubilizing functions in *O. ficus- indica* [64], but in *A. sisalana* has been identified as the main causal agent of “*bole rot*” [65]. Instead, *Bisifusarium* was found in high prevalence as root endophyte in both wild and traditional samples and previously reported as the causal agent of cladode “*soft rot*” in *Opuntia ficus-indica* [64] and *Nopalea cochenillifera* [66]. The differential abundance of specific fungal and prokaryotic pathogens in compartments and cultivated soils could be a response to conventional management practices in monoculture agaves, which promote high genetic homogeneity, allowing pathogens to develop strategies to evade plant defenses, as reported for prokaryotic communities in *A. tequilana* [2]. Surprisingly, even some Glomeromycota taxa, such as *Orientoglomus* and *Kamienskia*, showed differential abundance in the soils of wild populations compared to cultivated populations, a trend similar to that reported for *A. tequilana*, for which mycorrhizal taxa were absent from all samples, including soils [2]. The lack of mycorrhizal association has been linked to anthropogenic disturbances due to the introduction of non-native flora, such as monoculture agricultural practices [44], manifesting the effects of conventional agriculture on agave fungal communities.

### Management Practices and Microbial Communities in *A. angustifolia* Cultivation

Our findings highlight the profound impact of agricultural management practices on the diversity and composition of microbial communities in *A. angustifolia*. Traditional management practices, characterized by the use of seeds and vegetative propagules from diverse sources, sustain higher genetic and microbial diversity compared to conventional monoculture systems reliant on clonal propagation. These practices not only preserve microbial richness but also support beneficial functional traits within the microbiome, crucial for nutrient cycling, plant growth promotion, and pathogen resistance. In contrast, conventional systems show a marked reduction in microbial diversity, particularly in compartments such as the phyllosphere and rhizosphere, with potential functional implications. For instance, the depletion of Bacteroidetes, a group linked to polysaccharide digestion and plant growth under harsh conditions, may compromise plant resilience. Similarly, the prevalence of pathogens such as *Pantoea*, *Fusarium*, and *Aspergillus* in conventional systems underscores the vulnerabilities introduced by genetic homogeneity and management intensification.

The application of nitrogen fertilizers in conventional systems further alters microbial interactions, favoring nitrogen-fixing while disrupting ammonia-oxidizing archaea and promoting competitive microbial dynamics [60]. In contrast, traditional practices appear to recruit beneficial taxa, associated with biofertilization and pathogen suppression.

Management practices must prioritize strategies that foster microbial diversity and functionality across compartments. Since it has been demonstrated that the conversion of native vegetation to cropland promotes both changes in soil physicochemical properties (i.e., reductions in carbon storage, nutrient cycling, organic matter decomposition) and the depletion of beneficial fungal communities, while pathogenic increase in dominance as conventional agricultural practices are accentuated [67]. For example, reduced nitrogen inputs and the integration of biofertilizers could mitigate the negative impacts of conventional practices. Indeed, application of synthetic communities or SynComs designed based on the observed microbe-microbe interactions from wild agaves can increase microbial diversity and plant health and productivity in the cultivated *A. tequilana*, confirming that the microbiome of wild plants can be used for the benefit of the cultivated species. Additionally, promoting crop diversity through the cultivation of multiple agave varieties or intercropping can reduce pathogen pressure and enhance overall system resilience. For example, the maintenance of positive interactions among nurse and facilitator species in Agave mescal-producing systems are crucial to maintain phylogenetic diversity in these ecosystems, [68, 69] which, in the long-term, could maintain the “*eubiosis*” of the ecosystem (*i.e.,* by preserving the beneficial microbial communities). Therefore, the restoration of mycorrhizal associations, disrupted by monoculture practices, is critical for plant and soil health [23]. Mycorrhizal taxa, such as *Orientoglomus* and *Kamienskia*, play essential roles in nutrient acquisition and ecosystem stability. Thus, agroecological interventions, including organic amendments and reduced tillage, can help restore these beneficial relationships.

## Concluding Remarks

Overall, we found substantial evidence that the microbiome of *A. angustifolia* is shaped by environmental and management factors. At the environmental level, we found patterns previously described in agave species and plants from arid and semi-arid zones: prokaryotic communities are determined by humidity and temperature patterns, while fungal communities respond to biogeographic features that shape composition, possibly due to their lower dispersion. On the other hand, at the compartment level, there is evidence of genotype/phenotype selection in agaves, evidenced at the prokaryote level by a significant decrease of taxa of the phylum Bacteroidota, associated with holobiont health. At the fungal community level, Glomeromycota taxa are absent in soils from conventionally managed sites, manifesting the negative effect of pesticide use. Despite their incipient level of domestication, variations in the abundance and composition of the microbiome in the different plant compartments evidences a trend comparable to that described in species that have been under long periods of selection. The transition from intensive monoculture systems to diversified, traditional or agroecological approaches offers a pathway to enhance microbial diversity and functionality, ensuring more sustainable and resilient agave cultivation systems. Future studies should further explore the functional implications of microbial shifts and identify scalable practices for integrating microbiome management into agricultural systems.

## Supplementary Information

Below is the link to the electronic supplementary material.

Supplementary file1 (PDF 2957 KB)

Supplementary file2 (XLSX 33 KB)

## Data Availability

The raw sequencing data generated during this study are available in the NCBI Sequence Read Archive (SRA) under BioProject ID PRJNA1168991. All relevant data, including metadata and analysis scripts, will be made available upon reasonable request.
