# Supplementary material for: Agricultural Practices and Environmental Factors Drive Microbial Communities in the Mezcal-Producing Agave angustifolia Haw
Source: Microb Ecol. 2025 Jan 30;87(1):181. doi: 10.1007/s00248-025-02496-2 (PMC11779764; doi:10.1007/s00248-025-02496-2)
Supplement: Supplementary file 1 — Supplementary file1 (PDF 2957 KB) [file 248_2025_2496_MOESM1_ESM.pdf]

Agricultural practices and environmental factors drive microbial communities in the mezcal-producing *Agave angustifolia* Haw.

Gonzalo Contreras-Negrete[1*], Alfonso Valiente-Banuet[2], Francisco Molina-Freaner[2], Laila P. Partida-Martínez[3], and Antonio Hernández-López[1*]

[1]Ciencias Agrogenómicas, Escuela Nacional de Estudios Superiores Unidad León, Universidad Nacional Autónoma de México, León, Guanajuato, México
[2]Departamento de Ecología de la Biodiversidad, Instituto de Ecología, Universidad Nacional Autónoma de México, Mexico City, México
[3]Departamento de Ingeniería Genética, Centro de Investigación y de Estudios Avanzados del Instituto Politécnico Nacional, Unidad Irapuato, Irapuato, México

*Correspondence to: Antonio Hernández-López (ahernandez@enes.unam.mx) and Gonzalo Contreras-Negrete (congo@cieco.unam.mx) ORCID: 0000-0003-3713-4638

Supplementary table S2 Alpha diversities by collection site and compartment for the (a) prokaryotic and (b) fungal communities of *A. angustifolia*

a)

State	IAM	Status	Compartment	Chao1	Observed	Shannon
Sonora	AR	Conventional	Leave Endosphere	5.333	5.000	0.683
Sonora	AR	Conventional	Phyllosphere	1952.333	83.667	3.657
Sonora	AR	Conventional	Rhizosphere	8272.000	365.500	5.168
Sonora	AR	Conventional	Root Endosphere	141.333	79.000	4.046
Sonora	AR	Conventional	Soil	6269.000	171.000	5.030
Sonora	SAM	Conventional	Leave Endosphere	2.000	2.000	0.341
Sonora	SAM	Conventional	Phyllosphere	2371.000	62.000	3.346
Sonora	SAM	Conventional	Rhizosphere	2576.333	208.000	5.345
Sonora	SAM	Conventional	Root Endosphere	431.667	40.667	3.646
Sonora	SAM	Conventional	Soil	9966.000	124.000	4.626
Sonora	SAM	Traditional	Leave Endosphere	1.333	1.333	0.229
Sonora	SAM	Traditional	Phyllosphere	200.000	182.500	4.713
Sonora	SAM	Traditional	Rhizosphere	2012.000	316.333	4.882
Sonora	SAM	Traditional	Root Endosphere	185.000	87.000	2.322
Sonora	SAM	Traditional	Soil	4447.000	158.000	4.919
Sonora	SAM	Wild	Leave Endosphere	1.167	1.167	0.011
Sonora	SAM	Wild	Phyllosphere	400.000	127.500	4.048
Sonora	SAM	Wild	Rhizosphere	11779.000	289.500	5.031
Sonora	SAM	Wild	Root Endosphere	1040.667	70.667	3.913
Sonora	SAM	Wild	Soil	10834.000	193.000	5.083
Nayarit	HUM	Wild	Leave Endosphere	4.167	4.167	0.406
Nayarit	HUM	Wild	Phyllosphere	112.333	99.667	3.972
Nayarit	HUM	Wild	Rhizosphere	5164.500	163.500	5.343

Nayarit	HUM	Wild	Root Endosphere	1657.500	83.833	3.098
Nayarit	HUM	Wild	Soil	3709.500	202.500	5.109
Jalisco	HUM	Traditional	Leave Endosphere	1.000	1.000	0.000
Jalisco	HUM	Traditional	Phyllosphere	6233.000	208.000	5.081
Jalisco	HUM	Traditional	Rhizosphere	5746.000	239.333	5.327
Jalisco	HUM	Traditional	Root Endosphere	1208.000	127.000	4.087
Jalisco	HUM	Traditional	Soil	5829.000	159.000	4.873
Jalisco	SH	Traditional	Leave Endosphere	4.500	3.000	0.631
Jalisco	SH	Traditional	Phyllosphere	3460.000	141.667	4.332
Jalisco	SH	Traditional	Rhizosphere	2425.333	263.333	4.973
Jalisco	SH	Traditional	Root Endosphere	188.667	63.000	4.037
Jalisco	SH	Traditional	Soil	4815.000	163.000	4.849
Jalisco	HUM	Wild	Rhizosphere	12106.000	315.000	5.299
Jalisco	SH	Wild	Leave Endosphere	3044.000	2.600	0.556
Jalisco	SH	Wild	Phyllosphere	1562.000	86.333	3.587
Jalisco	SH	Wild	Rhizosphere	8215.667	206.000	4.999
Jalisco	SH	Wild	Root Endosphere	1972.667	88.667	3.945
Jalisco	SH	Wild	Soil	8336.000	164.000	4.946
Oaxaca	SH	Conventional	Leave Endosphere	2.000	2.000	0.374
Oaxaca	SH	Conventional	Rhizosphere	3194.000	242.000	5.074
Oaxaca	SH	Conventional	Root Endosphere	3095.667	107.000	3.710
Oaxaca	SH	Traditional	Leave Endosphere	3.000	3.000	0.376
Oaxaca	SH	Traditional	Phyllosphere	199.000	166.000	4.255
Oaxaca	SH	Traditional	Rhizosphere	5307.500	257.000	3.421
Oaxaca	SH	Traditional	Root Endosphere	1555.667	98.667	4.298

b)

State	IAM	Status	Compartment	Chao1	Observed	Shannon
Sonora	AR	Conventional	Leave Endosphere	282.000	24.000	3.156

Sonora	AR	Conventional	Root Endosphere	3097.333	76.333	4.101
Sonora	AR	Conventional	Phyllosphere	49.333	10.667	3.292
Sonora	AR	Conventional	Rhizosphere	816.000	39.000	3.043
Sonora	AR	Conventional	Soil	2485.000	70.000	3.807
Sonora	SAM	Conventional	Leave Endosphere	565.667	32.333	3.431
Sonora	SAM	Conventional	Root Endosphere	1052.000	43.000	3.658
Sonora	SAM	Conventional	Phyllosphere	12060.500	135.000	4.506
Sonora	SAM	Conventional	Rhizosphere	1246.000	48.333	4.255
Sonora	SAM	Conventional	Soil	3657.000	87.000	3.408
Sonora	SAM	Traditional	Leave Endosphere	374.000	24.000	2.806
Sonora	SAM	Traditional	Root Endosphere	708.667	36.333	2.949
Sonora	SAM	Traditional	Phyllosphere	10446.500	146.000	4.951
Sonora	SAM	Traditional	Rhizosphere	97.000	14.000	3.825
Sonora	SAM	Traditional	Soil	466.000	31.000	3.258
Sonora	SAM	Wild	Leave Endosphere	888.400	42.200	3.697
Sonora	SAM	Wild	Root Endosphere	2793.833	72.000	4.145
Sonora	SAM	Wild	Phyllosphere	5153.500	85.500	3.251
Sonora	SAM	Wild	Rhizosphere	1347.833	49.333	3.821
Sonora	SAM	Wild	Soil	716.500	36.500	3.860
Oaxaca	SH	Conventional	Leave Endosphere	524.000	34.500	3.425
Oaxaca	SH	Conventional	Root Endosphere	6764.333	116.000	4.653
Oaxaca	SH	Conventional	Rhizosphere	9872.000	142.000	4.322
Oaxaca	SH	Traditional	Leave Endosphere	436.000	29.667	3.350
Oaxaca	SH	Traditional	Root Endosphere	4939.000	95.667	4.299
Oaxaca	SH	Traditional	Phyllosphere	57.000	12.000	2.079
Oaxaca	SH	Traditional	Rhizosphere	4369.000	92.500	4.010
Nayarit	HUM	Wild	Leave Endosphere	332.833	23.167	2.760
Nayarit	HUM	Wild	Root Endosphere	524.333	25.167	2.792
Nayarit	HUM	Wild	Phyllosphere	401.667	21.667	3.072

Nayarit	HUM	Wild	Rhizosphere	2777.667	69.333	4.092
Nayarit	HUM	Wild	Soil	5570.500	106.000	4.577
Jalisco	HUM	Traditional	Leave Endosphere	485.000	29.667	3.310
Jalisco	HUM	Traditional	Root Endosphere	5443.333	99.000	4.055
Jalisco	HUM	Traditional	Phyllosphere	820.000	40.000	4.883
Jalisco	HUM	Traditional	Rhizosphere	5988.333	111.000	4.036
Jalisco	HUM	Traditional	Soil	253.000	22.000	4.419
Jalisco	SH	Traditional	Leave Endosphere	348.333	24.000	2.375
Jalisco	SH	Traditional	Root Endosphere	5807.000	110.000	4.647
Jalisco	SH	Traditional	Phyllosphere	2902.000	49.667	3.610
Jalisco	SH	Traditional	Rhizosphere	1397.333	42.000	2.665
Jalisco	SH	Traditional	Soil	21.000	6.000	4.248
Jalisco	HUM	Wild	Rhizosphere	4096.000	91.000	0.530
Jalisco	SH	Wild	Leave Endosphere	645.250	34.500	3.377
Jalisco	SH	Wild	Root Endosphere	6844.000	126.667	4.816
Jalisco	SH	Wild	Phyllosphere	6099.667	112.333	3.243
Jalisco	SH	Wild	Rhizosphere	957.000	36.333	4.114
Jalisco	SH	Wild	Soil	3486.000	83.000	1.792

Supplementary table S3.- PERMANOVA analysis of the microbial communities associated with *A. angustifolia,* at compartment level compartments considering, management status and IAM.

**Prokaryo**						**Fungi**					
**Compartment**	**Factor**	**D.F**	**F**	R^2	**P**	**Compartment**	**Factor**	**D.F**	**F**	R^2	**P**
**Leave Endosphe**						**Leave Endosph**					
	Stat	2	0.406	0.027	0.977		Stat	2	1.081	0.063	0.315
	IA	3	1.646	0.150	0.064		IA	3	1.821	0.150	**0.015**
**Phyllosph**						**Phyllosp**					
	Stat	2	1.443	0.145	**0.011**		Stat	2	1.040	0.109	0.361
	IA	3	1.076	0.160	0.248		IA	3	1.491	0.215	**0.010**
**Root Endosphe**						**Root Endosph**					
	Stat	2	1.128	0.064	0.201		Stat	2	1.149	0.065	**0.131**
	IA	3	1.385	0.113	**0.017**		IA	3	1.415	0.116	**0.001**
**Rhizosph**						**Rhizosph**					
	Stat	2	0.309	1.188	**0.047**		Stat	2	1.12	0.069	0.114
	IA	3	1.478	0.130	**0.0001**		IA	3	1.302	0.118	**0.003**
**Soil**						**Soil**					
	Stat	2	1.00	0.222	**0.45**		Stat	2	1.136	0.245	**0.03**
	IA	3	1.309	0.375	**0.007**		IA	3	1.012	0.319	0.423

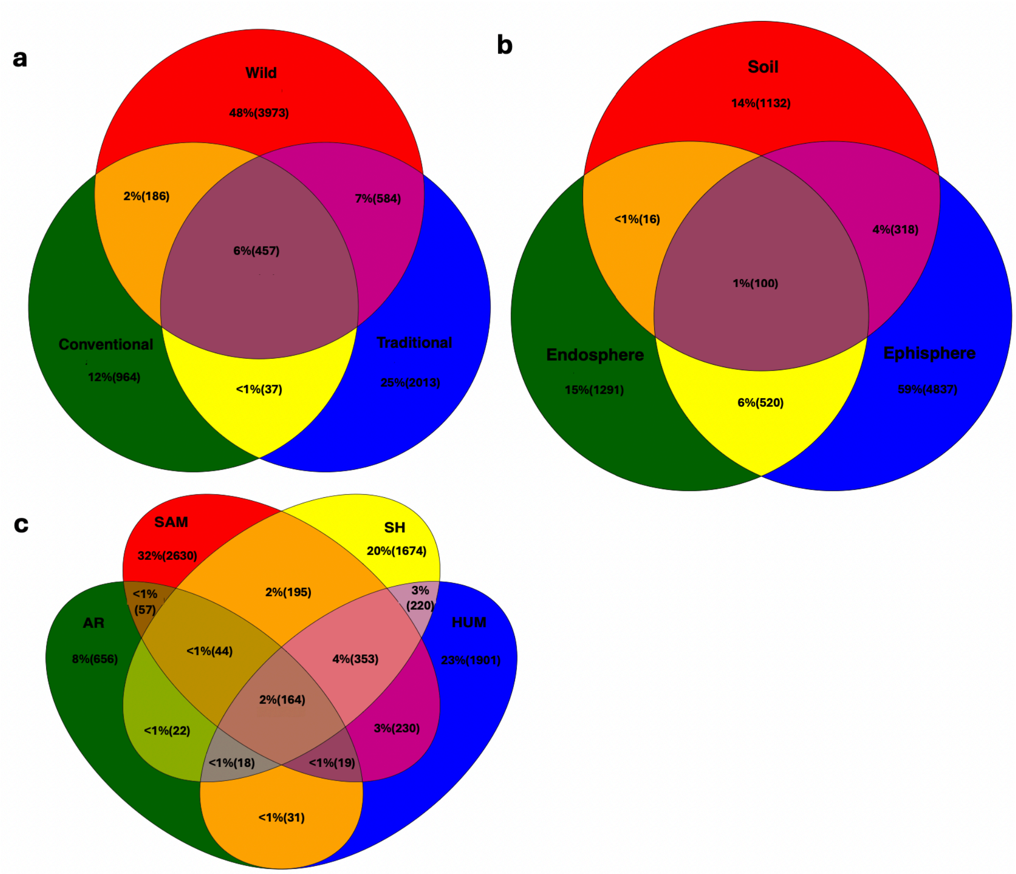

Supplementary figure S1.- Distribution of ASVs in different sample categories for prokaryotic communities in *A. angustifolia*. Venn diagrams show the number of exclusive and shared ASVs for: a) management status, b) sample type and c) IAM (De Martonne Aridity Index).

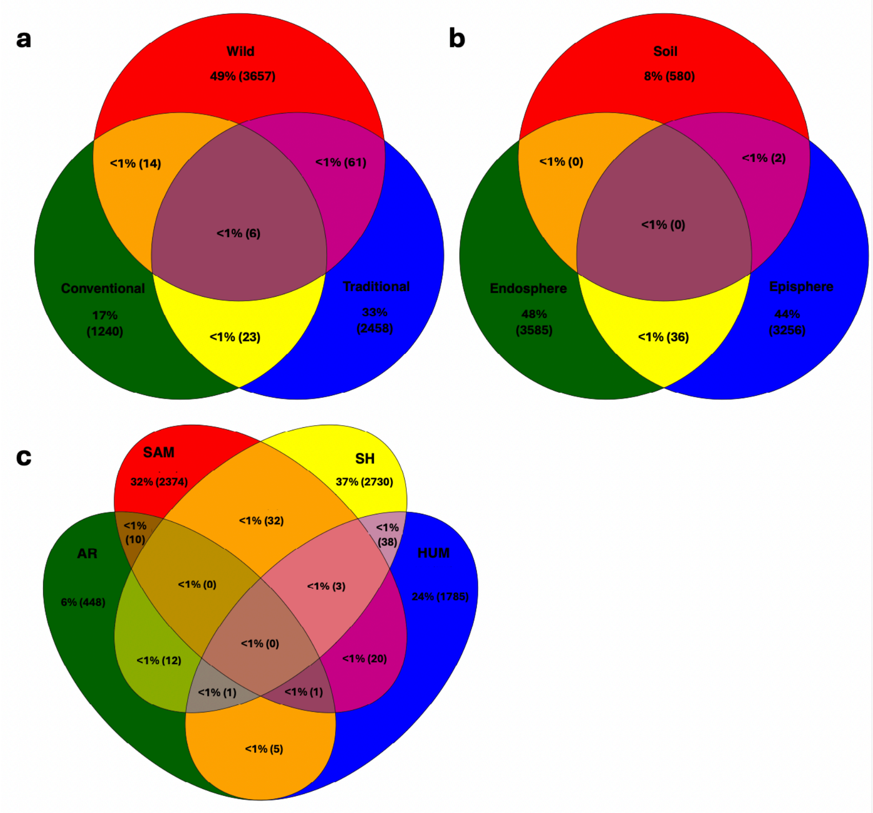

Supplementary figure S2.- Distribution of ASVs in different sample categories for fungal communities in *A. angustifolia*. Venn diagrams show the number of exclusive and shared ASVs for: a) management status, b) sample type and c) IAM (De Martonne Aridity Index).

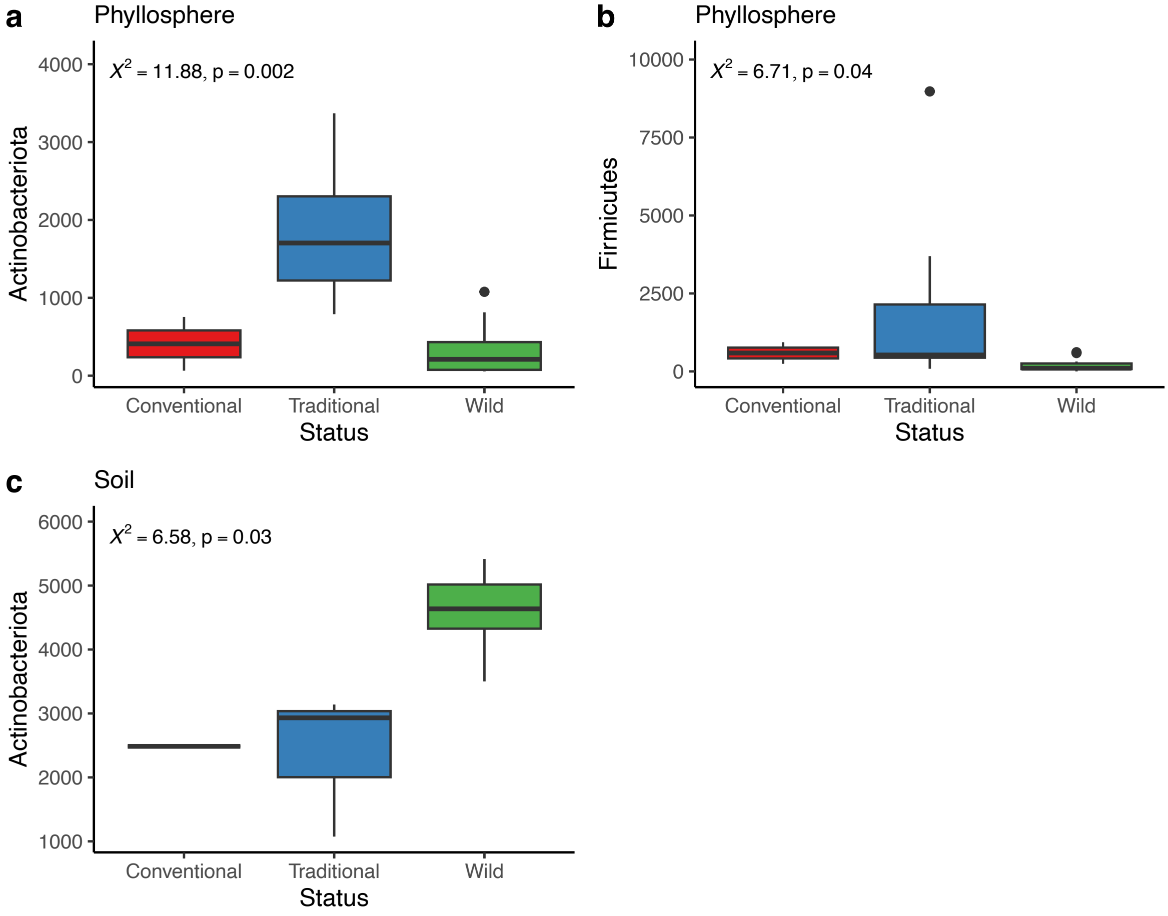

Supplementary figure S3.- Phylum-level differences in prevalence for prokaryotic communities under different management statuses: conventional, traditional and wild (*Kruskal-Wallis* X^2; p<0.05). Y axis shows the prevalence in reads for each prokaryotic Phylum.

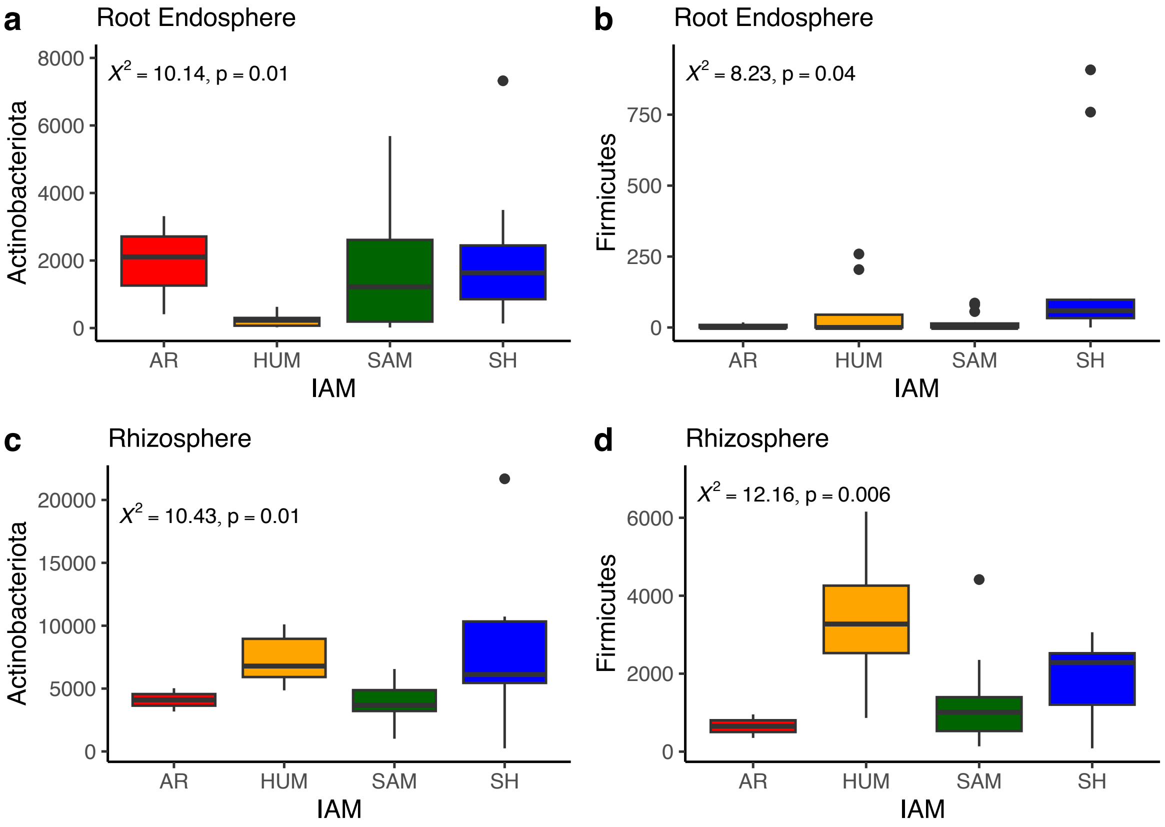

Supplementary figure S4.- Phylum-level differences in prevalence for prokaryotic communities under different IAM (De Martonne Aridity Index) (*Kruskal-Wallis* X^2; p<0.05). Y axis shows the prevalence in reads for each prokaryotic Phylum.

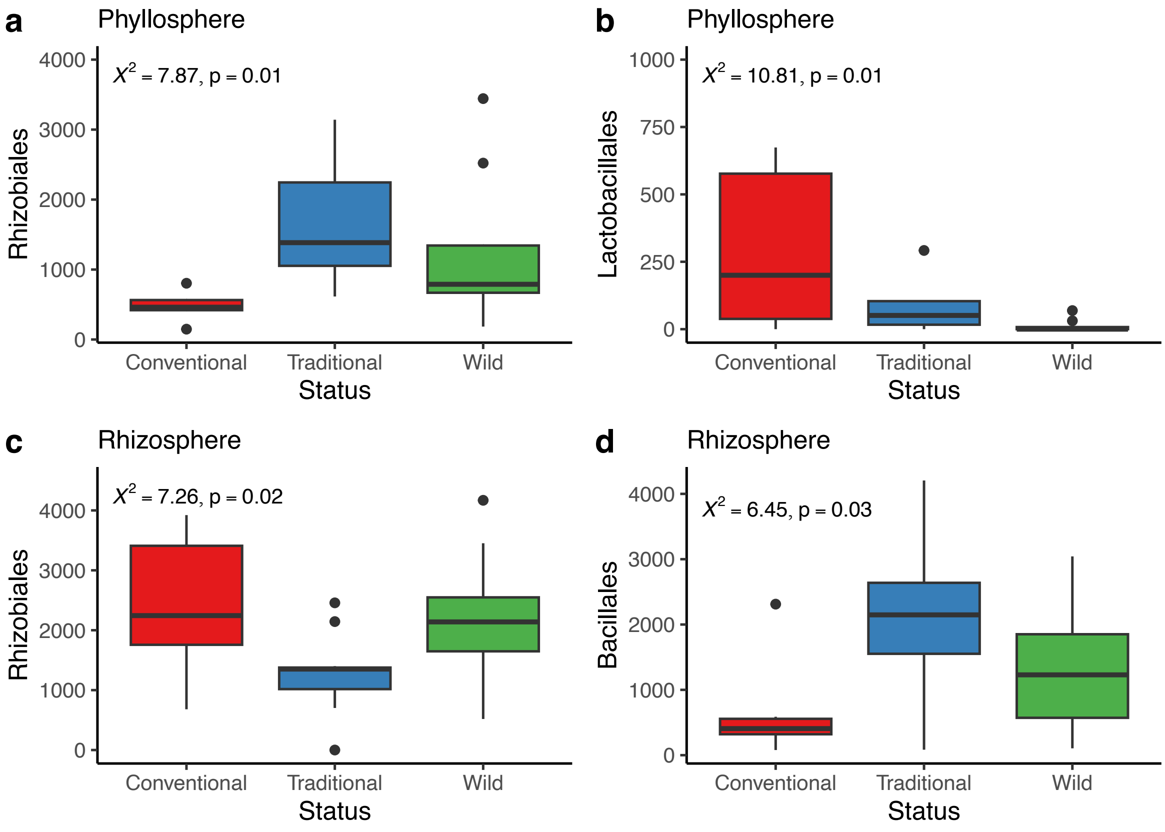

Supplementary figure S5.- Order-level differences in prevalence for prokaryotic communities under different management statuses: conventional, traditional and wild (*Kruskal-Wallis* X^2; p<0.05). Y axis shows the prevalence in reads for each prokaryotic Order.

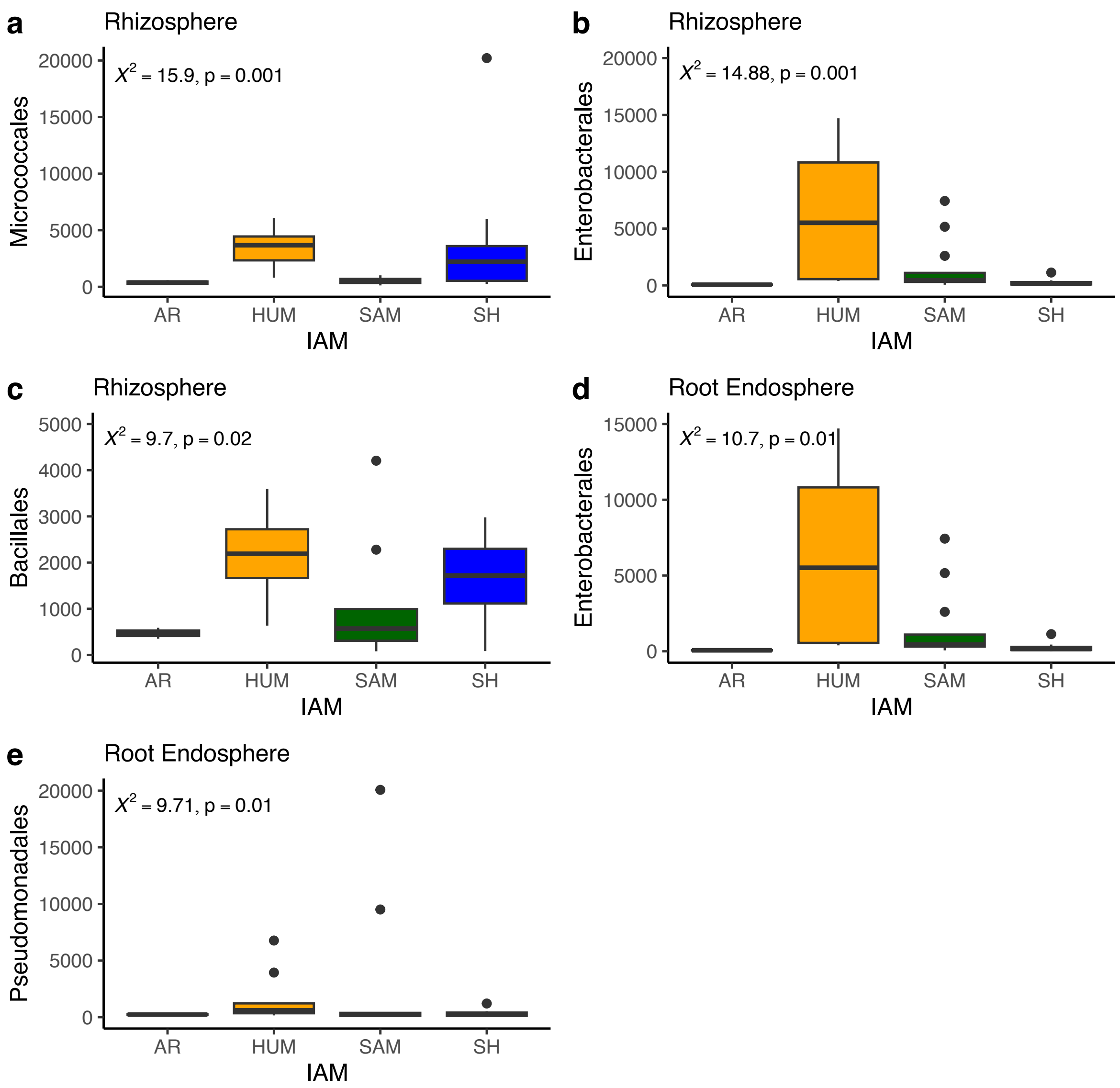

Supplementary figure S6.- Order-level differences in prevalence for prokaryotic communities under different IAM (De Martonne Aridity Index) (*Kruskal-Wallis* X^2; p<0.05). Y axis shows the prevalence in reads for each prokaryotic Order.

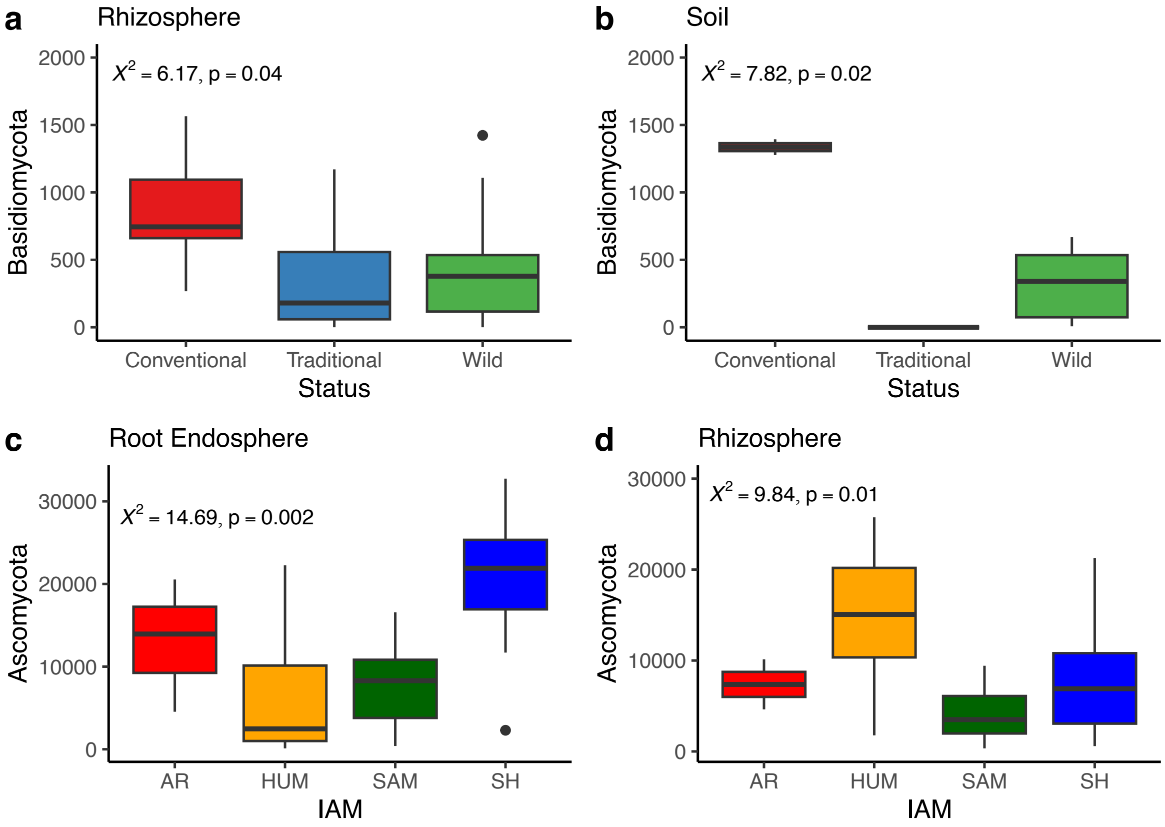

Supplementary figure S7.- Phylum-level differences in prevalence for fungal communities under different management statuses: conventional, traditional and wild (a and b) and under different IAM (De Martonne Aridity Index; c and d) (*Kruskal-Wallis* X^2; p<0.05). Y axis shows the prevalence in reads for each fungal phylum.

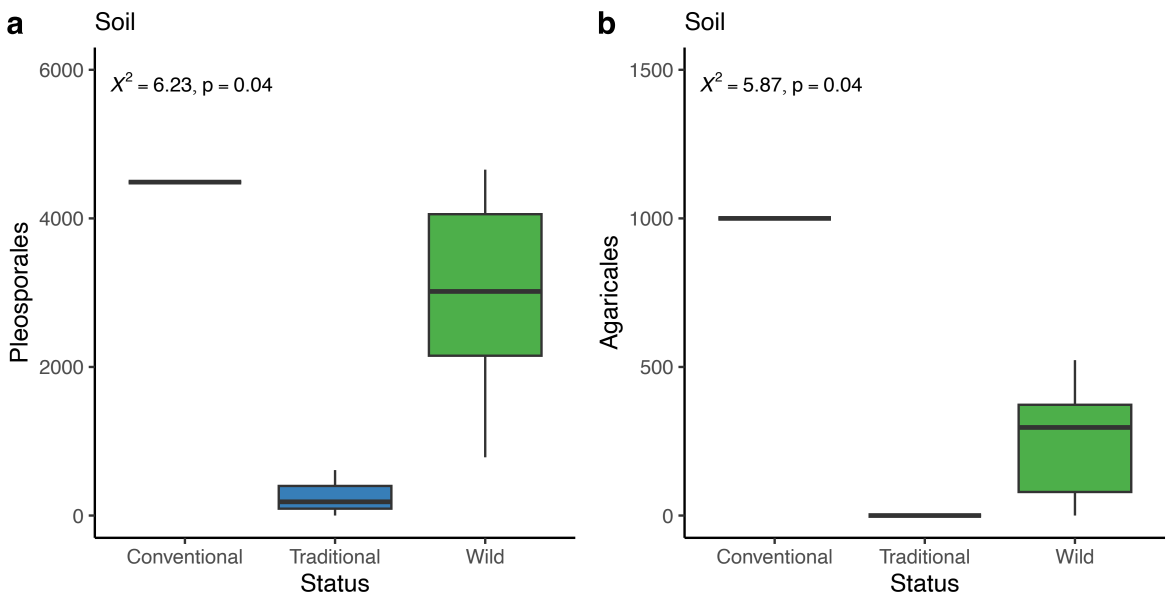

Supplementary figure S8.- Order-level differences in prevalence for fungal communities under different management statuses: conventional, traditional and wild (*Kruskal-Wallis* X^2; $p<0.05$). Y axis shows the prevalence in reads for each fungal Order.

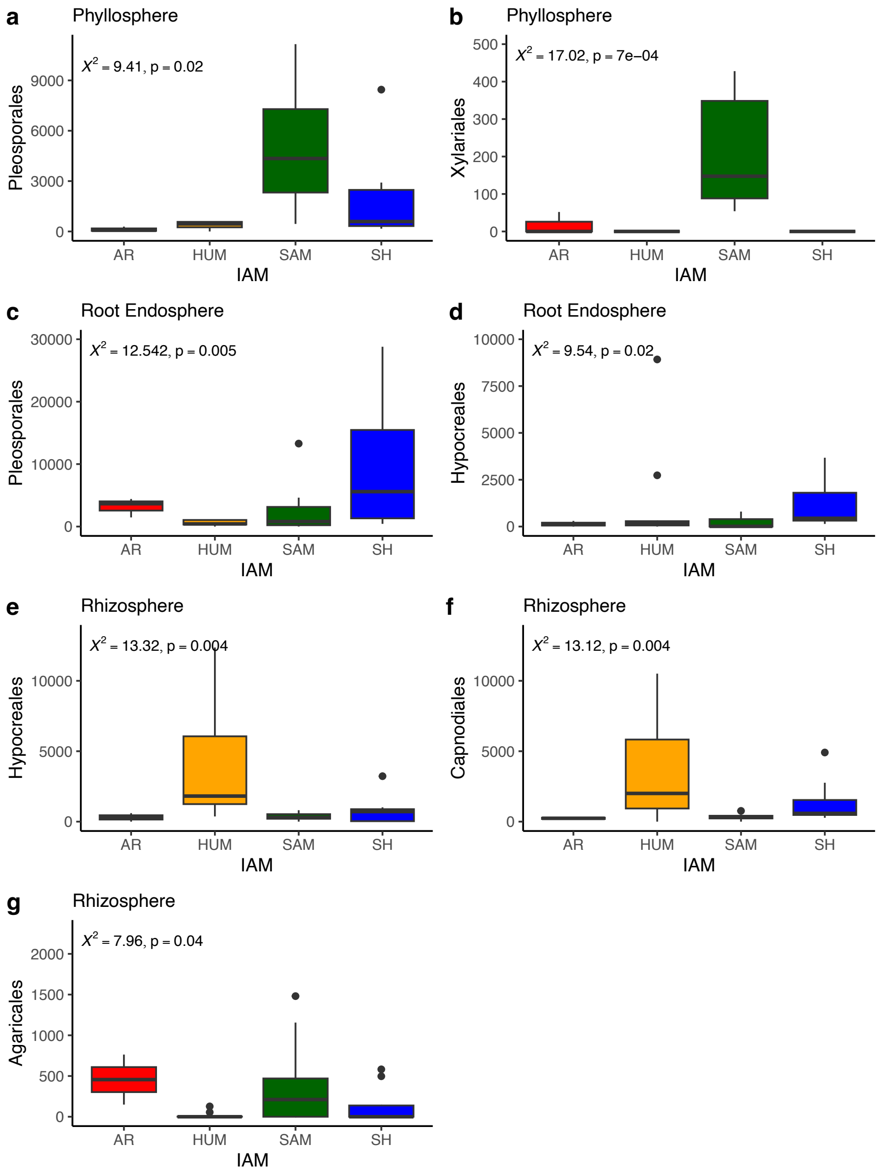

Supplementary figure S9.- Order-level differences in prevalence for fungal communities under different IAM (De Martonne Aridity Index) (*Kruskal-Wallis* X^2; p<0.05). Y axis shows the prevalence in reads for each fungal Order.

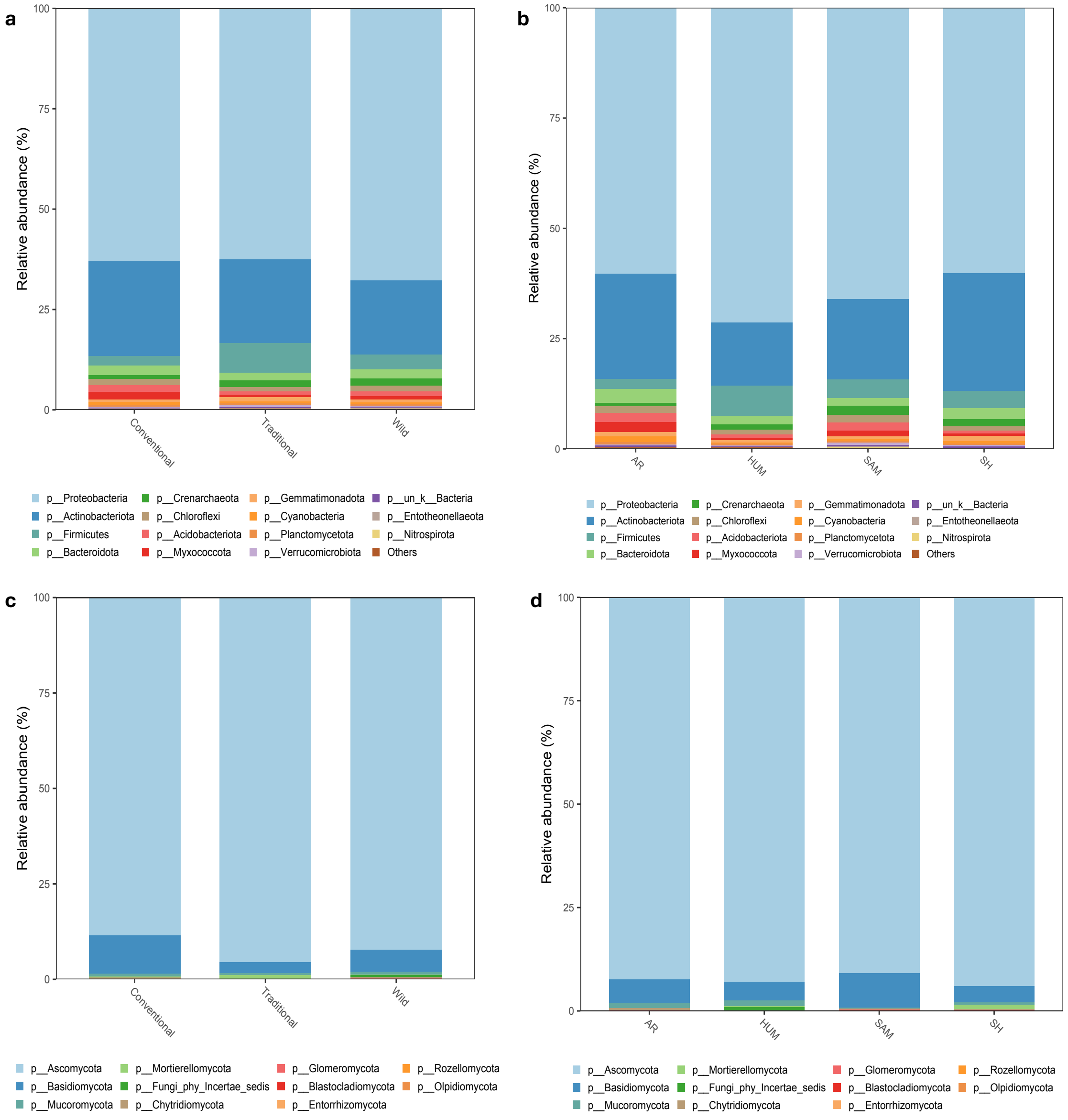

Fig S10.- Phylum level Major taxa players relative abundance for prokaryotic: a) status and b) IAM, and fungal: a) status and b) IAM communities

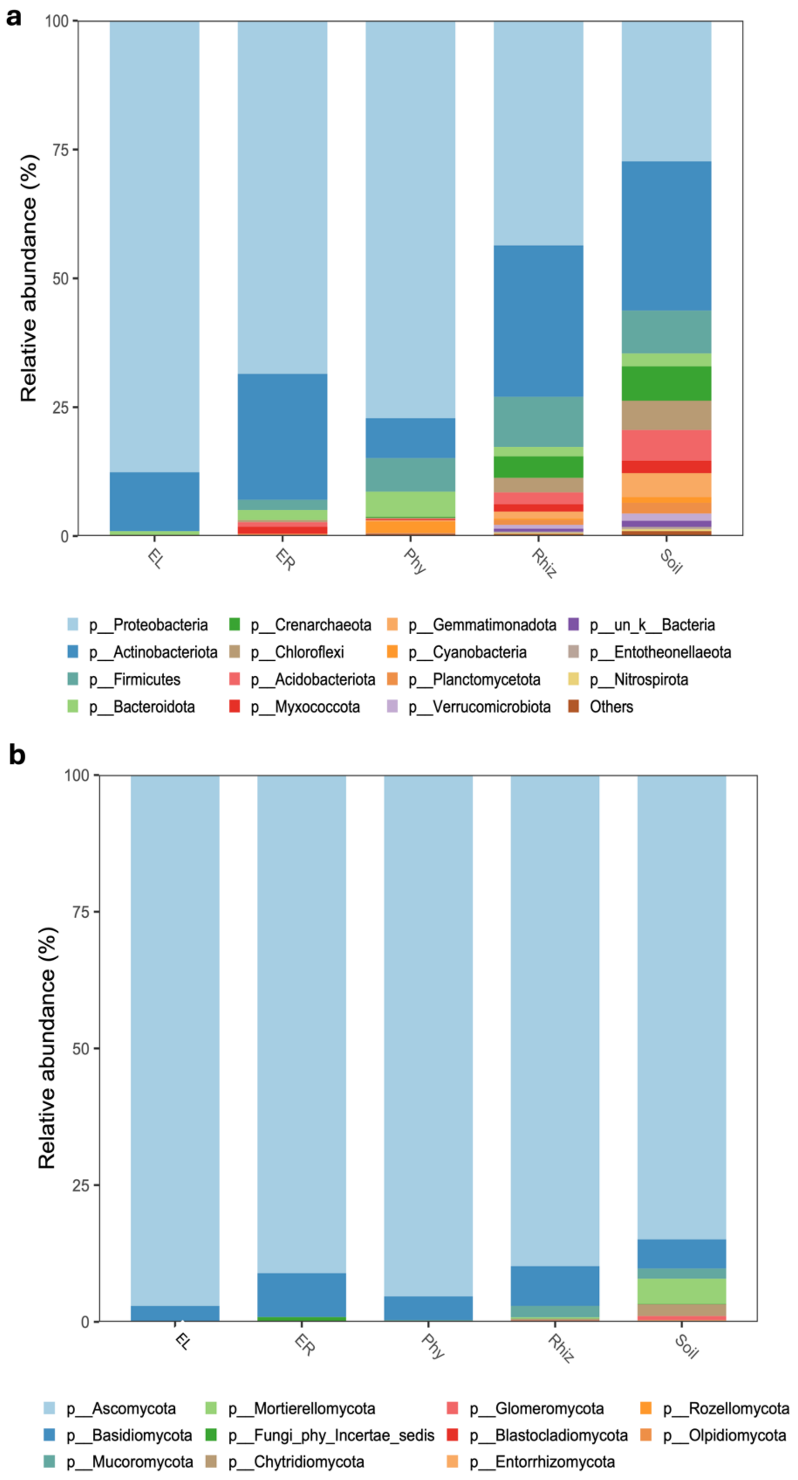

Fig S11.- Phylum level major taxa players relative abundance at compartment level for a) prokaryotic and b) fungal communities; EL= Leave Endosphere; ER= Root Endosphere; Phy= Phyllosphere; Rhi= Rhizosphere; Soil = Soil

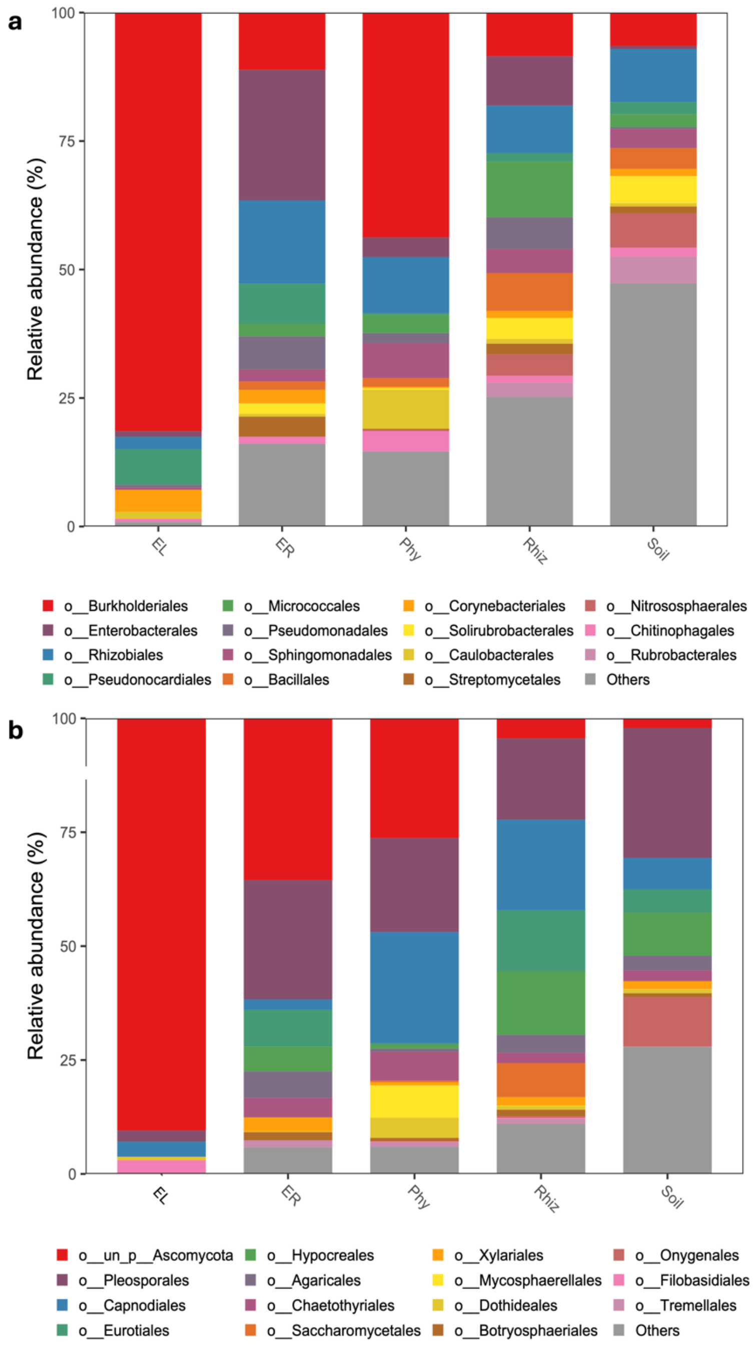

Fig. S12.- Order level major taxa players relative abundance at compartment level for a) prokaryotic and b) fungal communities; EL= Leave Endosphere; ER= Root Endosphere; Phy= Phyllosphere; Rhi= Rhizosphere; Soil = Soil

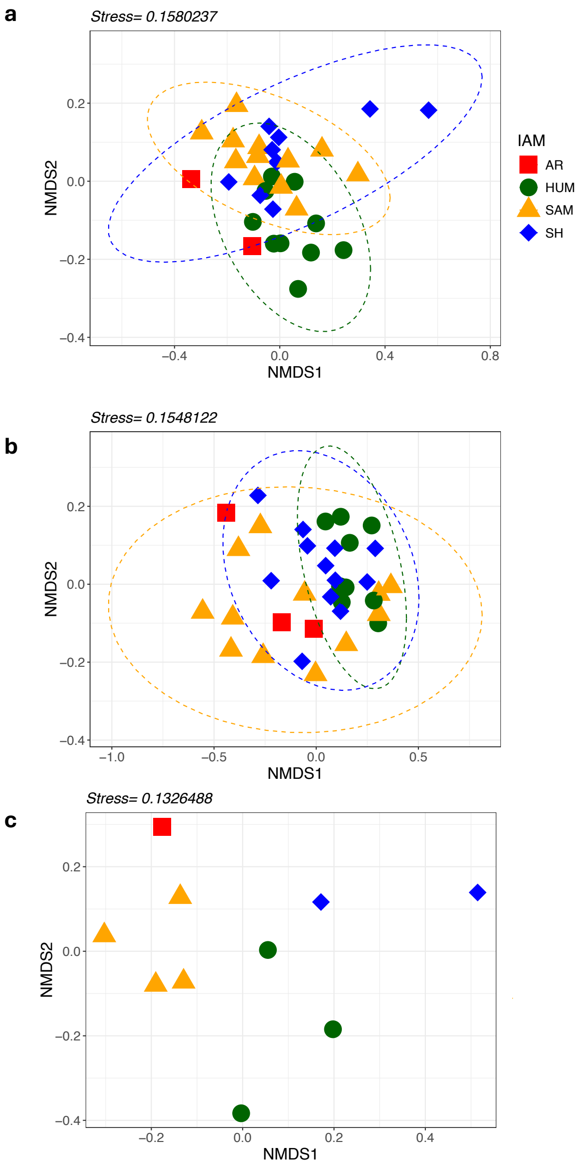

Fig S13.-Nonmetric multidimensional scaling (NMDS) plots for UniFrac distances of prokaryotic communities from *A. angustifolia* populations under different IAM (De Martonne Aridity Index): a) Rhizosphere IAM 16S, b) Root Endosphere and c) Soil. AR=Arid; SAM=Semiarid Mediterranean; SH= Subhumid; HUM=Humid

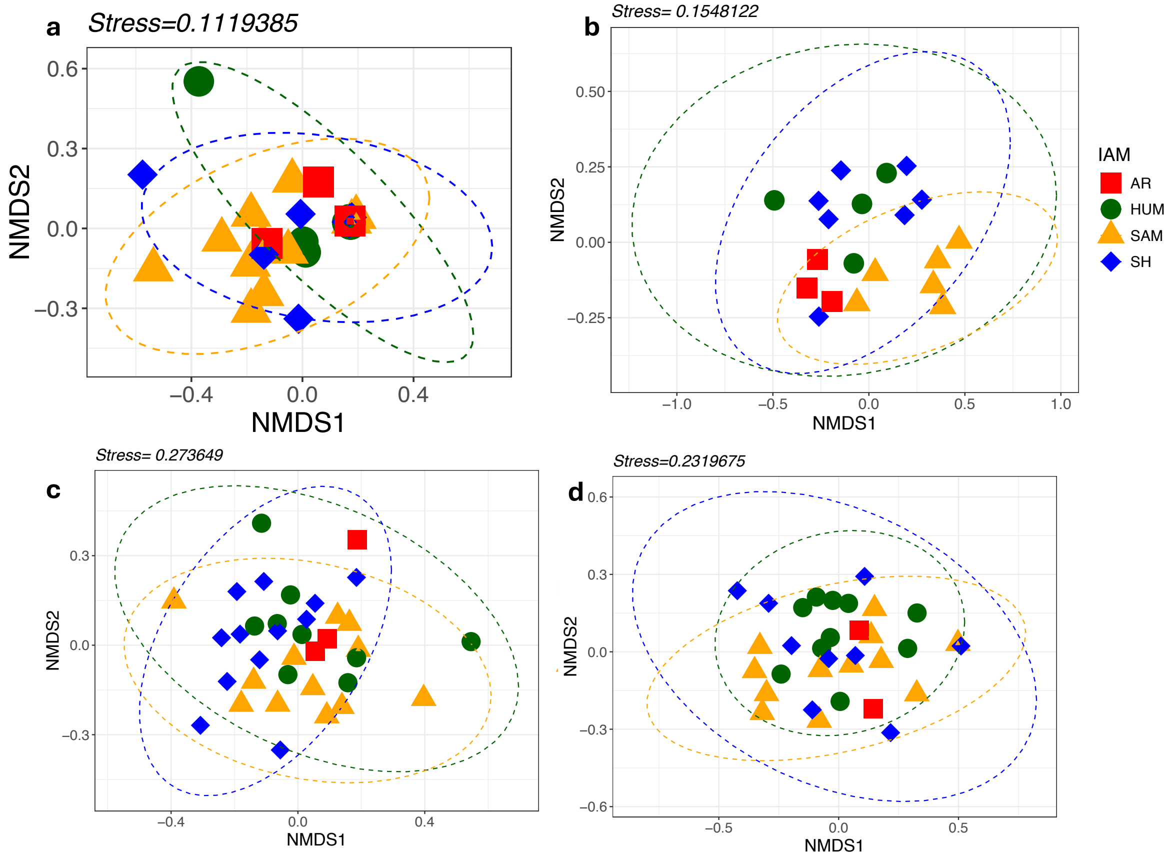

Fig S14.-Nonmetric multidimensional scaling (NMDS) plots for UniFrac distances of fungal communities from *A. angustifolia* populations under different IAM (De Martonne Aridity Index): a) Leaf Endosphere, b) Phyllosphere, c) Root Endosphere, d) Rhizosphere. AR=Arid; SAM=Semiarid Mediterranean; SH= Subhumid; HUM=Humid
